# Immune subtypes of megakaryocytes orchestrate inflammatory and regulatory responses to pulmonary infection via Treg crosstalk

**DOI:** 10.1172/JCI201621

**Published:** 2026-09-29

**Authors:** Huizhen He, Yezi Ma, Yifei Cai, Xiaoyuan Chen, Tianran Cheng, Ziqi Huo, Sibei Guo, Meijuan Xia, Dan Feng, Minmin Li, Jingjing Zhao, Nananan Zhao, Pei Su, Wen Zhou, Fei Wang, Cuicui Liu, Hongtao Wang, Jiaxi Zhou

**Affiliations:** 1 State Key Laboratory of Experimental Hematology, National Clinical Research Center for Blood Diseases, Haihe Laboratory of Cell Ecosystem, Institute of Hematology & Blood Diseases Hospital, Chinese Academy of Medical Sciences & Peking Union Medical College, Tianjin, China.; 2 Tianjin Institutes of Health Science, Tianjin, China.; 3 Haihe Laboratory of Cell Ecosystem, Tianjin Medical University, Tianjin, China.; 4 Cancer Research Institute, School of Basic Medical Science, Central South University, Changsha, Hunan, China.; 5 HaemoCure Inc, Tianjin, China.

**Keywords:** Cell biology, Hematology, Tregs

## Abstract

Recent studies have revealed that, beyond their classical role in platelet production, megakaryocytes (MKs) express immune-related genes and exert important immunoregulatory functions. However, it remains unclear whether these functions arise from a single versatile population or from distinct specialized subtypes, and how such subtypes influence infection and inflammation. Here, we identified 3 specialized immune MK subtypes (imm-MKs) — macrophage-like MKs (mac-MKs), neutrophil-like MKs (neu-MKs), and antigen-presenting MKs (APC-MKs) — each defined by distinct transcriptional programs and regulatory networks, with comparable heterogeneity observed in human MKs. Developmental analyses showed that MK immune-related programs increased with maturation and that immune MK subtypes exhibited distinct tissue- and stage-dependent patterns. Functionally, MK subtypes exhibited phase-specific responses during bacterial pneumonia: early infection preferentially induced mac-MKs and neu-MKs, which contributed to pulmonary inflammation, whereas during the postpeak acute-to-early recovery stage, APC-MKs supported a Treg-associated regulatory program that contributed to pulmonary inflammatory control. This MK-Treg axis uncovers a previously unrecognized mechanism of hematopoietic-immune crosstalk. Collectively, our study delineates organ- and stage-specific immune specialization of MKs and identifies immune MK subtypes as dynamic contributors to phase-specific inflammatory and Treg-linked regulatory programs during development and infection.

## Abstract 2

Megakaryocytes, the cells that make platelets, also help the immune system respond to lung infections. They can influence inflammation and work with regulatory T cells to help keep the immune response under control.

## Introduction

Megakaryocytes (MKs) are best known for their classical role in producing platelets, which are essential for maintaining hemostasis and blood coagulation (1–4). During early embryonic development, MKs first emerge in the yolk sac (5–8) and later become enriched in the fetal liver, which serves as the primary site of megakaryopoiesis during this stage (9, 10). After birth, the bone marrow gradually replaces the fetal liver as the main site of platelet production (11, 12), with the spleen and lung also contributing under certain physiological conditions (13–18).

Beyond platelet generation, MKs perform a wide array of additional functions. They actively shape the hematopoietic niche (19–23), support organ development (24, 25), and modulate immune responses (26). Through single-cell transcriptomic analysis and functional assays, recent studies—including our own—have revealed that MKs, across both embryonic and adult stages, exhibit remarkable cellular heterogeneity (27–33). Among these subtypes, platelet-MKs (plt-MKs) and mitochondrial-MKs (mt-MKs) are more closely associated with platelet production, niche-MKs with regulation of the hematopoietic niche, cycling-MKs (cyc-MKs) with proliferative or transitional states, and immune MKs with inflammatory or immune-associated activities (22, 33–37). Thus, MK subtypes may differ in both platelet-producing potential and functional specialization, rather than representing equivalent platelet-producing cells. These discoveries fundamentally expand our understanding of MK biology and underscore the importance of reevaluating their roles through the lens of cellular diversity (38–40).

Building on this concept, accumulating evidence now positions MKs as integral participants in immune regulation (41, 42). Similar to classical immune cells, MKs express a range of immune-related receptors and molecules — such as Toll-like receptors (TLRs), Fc receptors, cytokines, and chemokines — that equip them with immunosurveillance and regulatory capabilities (43). As our understanding of MK heterogeneity has deepened, it has become evident that their immune functions are primarily mediated by distinct immune subpopulations, which can be broadly identified by surface markers including CD53, CD48, and CXCR4 (28, 29, 32). Yet, it remains unresolved whether these immune MK subsets mirror the functional characteristics of traditional immune cells such as macrophages, neutrophils, or antigen-presenting cells. Furthermore, although immune-competent MKs have been observed at multiple developmental stages (44), how their immune properties evolve over time is still poorly understood.

Functionally, MKs have been reported to participate in both innate and adaptive immune responses (41). These MK-intrinsic immune functions are complemented by the immunoregulatory activities of their platelet progeny, including resting and aged/desialylated platelets, which have also been implicated in innate and adaptive immune processes (45–47). During innate immunity, they can recognize and phagocytose particulate matter — including fungi and bacteria — thereby contributing to early immune responses (43, 48, 49). In addition, they secrete CXCR2 ligands to promote neutrophil mobilization from the bone marrow (50), and release cytokines such as TNF-α and IL-6 to enhance neutrophil migration and bactericidal function (29). During adaptive immunity, MKs can present antigens via MHC class I molecules to activate CD8^+^ T cells and transfer these molecules to platelets. Platelets have also been shown to mediate MHC class I–restricted antigen presentation (51–53). MKs have the capacity to process and present antigens to CD4^+^ T cells, modulating downstream immune responses (49, 54, 55). Despite these insights, the mechanisms underlying such diverse immune functions remain incompletely defined — particularly whether they are executed by a single broadly immune-competent MK population or by multiple specialized subtypes acting in coordination.

In this study, we address these questions through single-cell transcriptomic profiling combined with in vivo functional analyses. We identify 3 distinct immune MK subtypes — macrophage-like MKs (mac-MKs), neutrophil-like MKs (neu-MKs), and antigen-presenting MKs (APC-MKs) — each defined by distinct transcriptional signatures and regulatory networks. Similar subtype architecture was observed in human MKs, and subtype-associated surface markers enabled downstream functional analyses. Developmental analyses showed that MK immune-related programs increased with maturation and that immune MK subtypes exhibited distinct tissue- and stage-dependent patterns. In a bacterial pneumonia model, early infection preferentially induced mac-MKs and neu-MKs, which contributed to early pulmonary inflammation. During the postpeak acute-to-early recovery stage, APC-MKs were linked to a Treg-associated regulatory program that supported pulmonary inflammatory control. Together, these findings reveal organ- and stage-specific immune specialization within the MK lineage and highlight MK subtypes as dynamic contributors to inflammatory and regulatory immune programs.

## Results

### Mouse megakaryocytes exhibit significant immune heterogeneity.

Our work, together with that of others, has demonstrated that megakaryocytes (MKs) are functionally heterogeneous, performing diverse roles in platelet production, microenvironmental support, and immune regulation through distinct subpopulations (27–31, 33). In both adult mouse and human bone marrow, we previously identified an immune-regulatory MK subpopulation (imm-MKs) (28). However, whether complex immune processes — such as phagocytosis, inflammatory modulation, and antigen presentation — are carried out by a single broadly immune-competent subset or by multiple functionally specialized MK subtypes remains unclear.

To address this, we conducted single-cell transcriptomic profiling (STRT-seq) of MKs isolated from bone marrow, spleen, and lung of adult mice (Figure 1A). After stringent quality filtering, we retained 867 high-quality MKs for downstream analysis (Supplemental Figure 1, A–C, and Supplemental Table 1A; supplemental material available online with this article; https://doi.org/10.1172/JCI201621DS1). Dimensionality reduction and clustering revealed 5 functionally distinct MK subpopulations — cyc-MKs, plt-MKs, mt-MKs, niche-MKs, and imm-MKs (Figure 1B, Supplemental Figure 1D, and Supplemental Table 2A) — in line with previous studies (28). The imm-MK cluster expressed numerous immune-related genes, including *Cd68*, *Retnlg*, and *Cd74*. Notably, the expression of these genes varied across cells within the group (Supplemental Figure 1E), suggesting that the imm-MK subpopulation may encompass additional layers of heterogeneity.

To further resolve this heterogeneity, we performed secondary clustering of the immune MK population (imm-MKs) and identified 3 distinct immune subtypes based on their characteristic immune signatures: macrophage-like MKs (mac-MKs), neutrophil-like MKs (neu-MKs), and antigen-presenting MKs (APC-MKs) (Figure 1C). All 3 subtypes retained expression of canonical MK markers (*Pf4*, *Itga2b*, *Ppbp*) and shared general immune genes (*Cd53*, *Nfkbia*, *Cd52*) (Figure 1D and Supplemental Figure 1F), yet each exhibited distinct transcriptional profiles consistent with different immune-related features. Mac-MKs exhibited macrophage-like features, including enrichment for phagocytosis and inflammation pathways and expression of markers like *Cd68* and *Lyz2*, along with high levels of *Mrc1* and *Atp6v1b2* (Figure 1, E–H). Neu-MKs showed neutrophil-like profiles, enriched for neutrophil activation pathways, and expressed *Retnlg*, *S100a9*, *Il1b*, and *Ngp*, indicating a potential role in acute inflammation. In contrast, APC-MKs exhibited features of antigen-presenting cells, with upregulation of *Cd74*, *Cd83*, *H2-Aa*, and *H2-Ab1*, alongside enrichment of antigen presentation pathways (Figure 1, E–H, Supplemental Table 2B, and Supplemental Table 3A).

To further distinguish these immune-associated MK subtypes from canonical immune cells, we compared mac-MKs, neu-MKs and APC-MKs with monocyte/macrophage, neutrophil, and APC populations, respectively. In the integrated scRNA-seq embedding, mac-MKs, neu-MKs and APC-MKs clustered separately from canonical immune-cell populations while retaining MK-lineage and MK maturation-associated gene programs (Supplemental Figure 1G). Although these MK subtypes expressed subsets of immune-related genes, their overall transcriptomic identities remained clearly distinct from canonical monocyte/macrophage, neutrophil, and APC clusters. Consistently, representative MK-lineage genes, including *Pf4*, *Itga2b*, and *Ppbp*, were expressed at higher levels in mac-MKs, neu-MKs, and APC-MKs than in canonical immune cells (Supplemental Figure 1H). Conversely, canonical immune-cell lineage markers were expressed at lower or more restricted levels in the corresponding MK subtypes than in their matched immune-cell populations, including *Cd68*, *Csf1r*, and *Tyrobp* for monocyte/macrophages; *Mmp8*, *S100a9*, and *Cd177* for neutrophils; and *H2-Ab1*, *H2-Aa*, and *Cd74* for APCs (Supplemental Figure 1H). Together, these analyses further confirm that mac-MKs, neu-MKs, and APC-MKs retain MK-lineage identity while acquiring partial immune-related transcriptional features, supporting their designation as immune-associated MK subtypes rather than canonical immune-cell populations.

To explore transcriptional regulatory programs associated with these immune features, we applied pySCENIC analysis to infer transcriptional regulatory networks (Figure 1I). Mac-MKs were enriched for macrophage-related transcription factors *Junb* and *Egr2 (*56, 57), neu-MKs showed increased activity of neutrophil-related regulons *Cebpe* and *Klf5* (58, 59), and APC-MKs expressed antigen presentation regulators *Pax5* and *Runx3* (60, 61) (Figure 1I). While all subtypes shared core MK regulators (*Gata1*, *Meis1*, *Fli1*) (62–64), their immune-related features were accompanied by distinct transcriptional regulatory programs.

In summary, our single-cell transcriptomic analysis of mouse MKs across bone marrow, spleen, and lung reveals pronounced immune heterogeneity within the imm-MK compartment. This subpopulation comprises 3 immune MK subtypes — mac-MKs, neu-MKs, and APC-MKs — each defined by distinct gene expression patterns and regulatory features. Together, these findings reveal previously unrecognized transcriptional heterogeneity within the immune MK compartment.

### Identification of distinct immune megakaryocyte subtypes.

To validate the presence of the immune MK subtypes identified by transcriptomic profiling, we systematically searched for subtype-enriched surface markers of mac-MKs, neu-MKs, and APC-MKs using single-cell transcriptomic data. Candidate genes were selected based on 2 criteria: (a) encoding membrane or cell-surface proteins, and (b) enriched expression in the corresponding transcriptomic subtype. Through this screening, we identified *Adgre1* (F4/80) for mac-MKs, *Ly6g* (Ly6G) for neu-MKs, and *H2-Aa* (MHCII) for APC-MKs as promising markers (Figure 2A). Violin plots showed enriched expression of *Adgre1*, *Ly6g*, and *H2-Aa* in their corresponding subtypes (Figure 2B).

We next validated these candidate markers using immunofluorescence staining, imaging flow cytometry, and conventional flow cytometry on MKs from bone marrow, spleen, and lung. Immunofluorescence staining showed that F4/80, Ly6G, and MHCII signals could be detected in CD41^+^ MKs across these tissues (Figure 2, C–E). To further visualize these marker-positive events at the single-cell level, we performed imaging flow cytometry. In bone marrow, spleen, and lung, representative CD41^+^ CD42c^+^ MKs events showed detectable F4/80, Ly6G, or MHCII signals (Figure 2, F–H). We then quantified these populations using conventional flow cytometry. Representative gating plots showed that F4/80, Ly6G, and MHCII expression was detected within the CD41^+^ CD42c^+^ MKs gate (Supplemental Figure 2, A–C). In the bone marrow, F4/80^+^ mac-MKs, Ly6G^+^ neu-MKs, and MHCII^+^ APC-MKs accounted for 7.08% ± 0.49%, 7.11% ± 0.68%, and 6.21% ± 0.61% of total bone marrow MKs, respectively (Figure 2I). In the spleen, these 3 immune MK subtypes were also detected. Notably, compared with the bone marrow, the proportion of Ly6G^+^ neu-MKs in the spleen was markedly reduced, whereas that of MHCII^+^ APC-MKs was significantly elevated (Figure 2I). Similarly, these 3 immune MK subtypes were also present in the lung, accounting for 5.27% ± 0.37% F4/80^+^ mac-MKs, 0.38% ± 0.05% Ly6G^+^ neu-MKs, and 11.16% ± 0.77% MHCII^+^ APC-MKs of total lung MKs, respectively (Figure 2I). Collectively, these findings demonstrate that mac-MKs, neu-MKs, and APC-MKs are broadly distributed across MK-resident organs, with distinct organ-specific variations in their relative abundance.

To further explore whether the identified immune MK subtypes differed in their association with Flt3-lineage history across tissues, we used Flt3^Cre^; Rosa26^tdTomato^ mice, a lineage-tracing model previously used to distinguish Flt3/Flk2-lineage–traced and –untraced MK populations (14, 65). In this model, tdTomato^+^ and tdTomato^–^ MKs represent Flt3-lineage–traced and –untraced MKs, respectively (Supplemental Figure 2D). We found that, in adult bone marrow and spleen, total MKs and the 3 subtypes were predominantly enriched in the tdTomato^+^ fraction, whereas lung MKs showed a higher tdTomato^–^ contribution, particularly within F4/80^+^ and MHCII^+^ MKs (Supplemental Figure 2E). These results suggest that immune MK subtypes can also be traced using a genetic approach and that their association with the Flt3 lineage differs across tissues.

In summary, F4/80, Ly6G and MHCII were identified as subtype-associated surface markers for mac-MKs, neu-MKs, and APC-MKs, respectively. By combining these identified surface makers with a lineage-tracing genetic approach, we were able to reliably identify and characterize 3 immune MK subtypes in bone marrow, spleen and lung.

### Human megakaryocytes exhibit similar immune heterogeneity.

Having established pronounced immune heterogeneity among mouse MKs, we next examined whether similar features are conserved in humans. To this end, we reanalyzed published single-cell transcriptomic datasets of human MKs derived from fetal liver and adult bone marrow (27, 28) (Figure 3A and Supplemental Table 1B). Subclustering of the human imm-MK population revealed 3 distinct subtypes that closely resembled the mouse mac-MKs, neu-MKs, and APC-MKs profiles (Figure 3B and Supplemental Table 2C).

Specifically, the human mac-MKs subtype exhibited elevated macrophage signature scores, enrichment in phagocytosis-related pathways, and upregulation of *mannose receptor C-type 1 (MRC1)* and *apolipoprotein E* (*APOE*). The neu-MKs subtype displayed the highest neutrophil signature scores and was enriched for neutrophil activation genes, including *CXCL1* and *DEFA3*. The APC-MKs subtype showed enhanced expression of antigen presentation markers such as *HLA-DRB5* and *HLA-DQB1*, along with enrichment of T cell antigen processing pathways (Figure 3, C–E, and Supplemental Table 3B).

We further observed developmental stage–specific differences in subtype composition: fetal liver MKs were predominantly mac-MKs, whereas adult bone marrow contained all 3 subtypes (Figure 3F). Surface marker screening identified *CD163* (mac-MKs), *CD177* (neu-MKs), and *HLA-DRA* (APC-MKs) as subtype-enriched candidate markers (Figure 3G). Flow cytometry confirmed their presence in adult human bone marrow, identifying mac-MKs (0.68% ± 0.07%), neu-MKs (6.77% ± 0.50%), and APC-MKs (5.64% ± 0.72%) (Figure 3H). To explore whether those subtypes have any functional potential, we coincubated human bone marrow primary cells with GFP^+^ *E*. *coli* and then analyzed GFP-positive bacterial signals within the CD41a^+^ CD42b^+^ human MKs gate (Supplemental Figure 3A). Interestingly, we found CD163^+^ MKs showed a higher frequency of GFP^+^ events than CD163^–^ MKs. These results indicate that human Mac-MKs also possess a phagocytic-like functional potential (Supplemental Figure 3B).

In summary, human MKs exhibit quite similar immune heterogeneity, comprising mac-MKs, neu-MKs, and APC-MKs that parallel with murine subtypes. These subtypes retain distinct immune-related transcriptional features — macrophage-associated, neutrophil-associated, and antigen-presentation–related programs — and display developmental differences between fetal liver and adult bone marrow.

### Temporal remodeling of immune megakaryocyte subtypes during development.

Building on our cross-species analyses, we next examined how MK immune specialization evolves during development. The differences between embryonic and adult tissues suggested that immune MK subtypes may arise or diversify as development proceeds. To test this, we generated single-cell transcriptomic atlases of MKs from neonatal (P7) and adult (8W) mice in the bone marrow and spleen, 2 key hematopoietic organs, after birth. Using STRT-seq, we profiled MKs from P7 bone marrow and spleen, and after rigorous quality control and integration with adult datasets, obtained 1,003 high-quality cells for downstream analysis (Figure 4A, Supplemental Figure 4A, and Supplemental Table 1A). Immune scoring revealed that neonatal MKs exhibited low immune-related signature activity, whereas adult MKs showed markedly elevated immune signatures, suggesting a developmental enhancement of immune-related transcriptional programs (Supplemental Figure 4, B and C, and Supplemental Table 2A). This developmental pattern is consistent with prior evidence of age-dependent differences in platelet function and platelet-mediated immune regulation (66, 67). Consistently, the immune MK subpopulation was substantially more abundant in adult bone marrow and spleen than in neonatal samples, indicating progressive enrichment of immune-specialized MKs during maturation (Supplemental Figure 4, B and C).

In bone marrow, mac-MKs, neu-MKs, and APC-MKs were more frequently represented in adult than neonatal samples based on subtype annotation and flow cytometry validation (Figure 4, B and C, and Supplemental Figure 4D). In the spleen, mac-MKs and APC-MKs frequencies increased over time, while neu-MKs representation showed a slight decline (Figure 4D). To validate these transcriptomic trends, we used flow cytometry to quantify immune MK subtypes across developmental stages. Consistently, mac-MKs, neu-MKs, and APC-MKs were detectable in the bone marrow at P7, with an average proportion below 2%, and increased to nearly 10% by 8W (Figure 4C). In the spleen, mac-MKs and APC-MKs gradually increased from P7 to 8W, whereas neu-MKs remained very low throughout, decreasing from approximately 1% at P7 to 0.1% at 8W (Figure 4D). Collectively, these data indicate developmental remodeling of immune MK subtypes, with distinct tissue-specific dynamics across subtypes.

To further explore MK immune heterogeneity in a distinct hematopoietic context, we extended our analysis to the lung — a unique site of extramedullary hematopoiesis and immune specialization (10, 41, 56, 57). We generated a developmental single-cell atlas of lung MKs across embryonic (E13.5, E15.5, E17.5), neonatal (P1) and adult (8W) stages. After stringent quality control and integration, 971 high-quality lung MKs were retained for analysis (Figure 4E, Supplemental Figure 4E, and Supplemental Table 1A). Principal component analysis revealed clear segregation of embryonic, neonatal, and adult MKs, indicating substantial developmental transcriptional shifts (Supplemental Figure 4F). Immune scoring showed minimal immune activity in embryonic MKs, which progressively increased postnatally and peaked in adulthood, indicating a developmental trajectory toward enhanced immune specialization (Figure 4F).

Immune-associated transcriptional programs became more evident during postnatal lung MK development, whereas platelet-producing and niche-associated transcriptional programs declined after birth (Supplemental Figure 4G). These findings indicate postnatal enrichment of immune-associated transcriptional features in adult lung MKs. Subclustering of the imm-MK population identified mac-MK, neu-MK, and APC-MK subtypes, mirroring those in bone marrow and spleen (Figure 4G and Supplemental Figure 4H). Each displayed distinct temporal dynamics: mac-MKs emerged during embryogenesis and expanded rapidly postnatally, whereas neu-MKs and APC-MKs appeared mainly in adulthood (Figure 4H). Flow cytometry analysis confirmed a progressive increase in mac-MKs during lung development, rising from 0.41% ± 0.05% at E15.5 to 3.45% ± 0.23% at P1, and further to 5.44% ± 0.23% at 8W (Figure 4I). In contrast, neu-MKs and APC-MKs remained low at both E15.5 and P1 (0.1%–0.2%) and only showed a marked increase at 8W (Figure 4I).

Together, our developmental atlases of MKs from bone marrow, spleen, and lung reveal progressive strengthening of immune function accompanied by distinct temporal and tissue-specific dynamics among immune MK subtypes. These findings illuminate the molecular and cellular programs associated with MK immune specialization during development.

### Mac-MKs and neu-MKs contribute to early pulmonary inflammation during bacterial pneumonia.

The discovery of temporal- and tissue-specific immune specialization among MK subtypes prompted us to investigate whether these molecularly distinct subtypes exert discrete immune functions. Although transcriptional profiling revealed developmental expansion and subtype heterogeneity of immune MKs, their in vivo roles during infection remained unclear. The lung, which exhibits pronounced enrichment of immune MKs in adulthood, provided an ideal context to examine their function under physiological challenge.

To delineate subtype-specific responses during infection, we used an *Escherichia coli* (*E*. *coli*) pneumonia model to examine the dynamic roles of MK subtypes in host immune responses (Supplemental Figure 5A). Pulmonary bacterial burden and inflammatory indices, including IL-6 and TNF-α in bronchoalveolar lavage fluid (BALF), as well as pulmonary neutrophil accumulation, peaked on day 1 after infection, followed by a marked decrease by day 4 and a further numerical decrease on days 7 and 10; however, the decreases observed on days 7 and 10 did not reach statistical significance (Supplemental Figure 5, B–E). Consistently, gross lung morphology and H&E staining revealed severe pulmonary inflammation on day 1, followed by partial histological improvement by day 4 (Supplemental Figure 5F). These kinetics defined day 1 as the peak acute inflammatory stage and day 4 as the after-peak acute-to-early-recovery transition, providing a framework for assessing stage-specific responses of MK subtypes.

In 8–12-week-old adult mice, flow cytometric analysis revealed marked expansion of mac-MKs and neu-MKs at day 1 postinfection, whereas APC-MKs were not significantly increased at this stage (Figure 5A). Interestingly, we found that the total number of lung MKs did not increase significantly within 24 hours after infection, despite the accumulation of F4/80^+^ and Ly6G^+^ MKs within the existing lung MK pool during this early period (Supplemental Figure 5, G–I). These findings suggest that the early expansion of immune MK subtypes may occur at the expense of other MK subtypes, reflecting a rapid shift in the composition of the resident lung MK subpopulation.

Given their selective induction during early infection, we next examined whether mac-MKs and neu-MKs exhibited functional properties relevant to the early pulmonary inflammatory response. Transcriptomic profiling showed that mac-MKs highly expressed phagocytosis-associated genes, including *Mrc1* and *Atp6v1b2*, implicating them in phagocytosis-related programs (Figure 1H). Consistently, in a GFP-labeled *E*. *coli* infection model, flow cytometry detected GFP-positive bacterial signals within F4/80^+^ MKs, with a significantly higher GFP^+^ frequency in F4/80^+^ MKs than in F4/80^–^ MKs (7.01% ± 1.05% versus 0.43% ± 0.15%, *P* = 0.0034), supporting that a subset of F4/80^+^ mac-MKs display phagocytic-like potential (Figure 5, B and C). In contrast, neu-MKs exhibited elevated expression of proinflammatory and antimicrobial genes such as *Il1b* and *Ngp* (Figure 1H). Consistent with these signatures, in vitro bacterial killing assays demonstrated that neu-MKs possessed significantly greater *E*. *coli* killing capacity than Ly6G^–^ MKs, supporting enhanced bactericidal potential of neu-MKs in vitro (Figure 5, D and E).

To further measure the in vivo function of lung MKs during early infection, we used PF4^Cre^-iDTR mice combined with oropharyngeal DT administration to deplete lung MKs (Supplemental Figure 5J). We found that oropharyngeal DT treatment effectively reduced lung MK proportions in PF4^Cre^-iDTR mice, but did not significantly affect bone marrow or splenic MK proportions (Supplemental Figure 5, K and L). We also found that oropharyngeal DT administration itself did not significantly alter the major lung immune-cell populations examined, including Ly6G^+^ neutrophils, F4/80^+^ macrophages, CD3^+^ T cells, and CD19^+^ B cells, in WT mice (Supplemental Figure 5, M–P). These observations indicate that our strategy provides a selective and effective approach for investigating the functions of lung MKs while exerting minimal effects on other immune-cell populations and MKs residing in other organs.

We then applied this strategy in the *E*. *coli* pneumonia model to examine the consequences of depleting lung MKs during the early inflammatory stage (Figure 5F). At day 1 after infection, DT treatment reduced total lung MKs as well as F4/80^+^ and Ly6G^+^ MK populations (Figure 5G). This reduction was accompanied by attenuated histological inflammation and decreased pulmonary neutrophil accumulation (Figure 5, H and I). These findings indicate that the early infection-induced lung MK compartment, particularly mac-MKs and neu-MKs, contributes to pulmonary inflammation during the acute phase of *E*. *coli* pneumonia.

### APC-MKs promote resolution of pulmonary inflammation via Treg regulation.

Having examined mac-MKs and neu-MKs responses during early infection, we next investigated MK subtype dynamics during the acute-to-early recovery stage. At day 4 after infection, the proportion of APC-MKs increased significantly, whereas mac-MKs and neu-MKs returned toward baseline levels (Figure 6A), suggesting that APC-MKs may be linked to late regulatory responses.

Regulatory T (Treg) cells suppress excessive inflammation and support tissue repair during pneumonia recovery (68). Given previous evidence that lung MKs can modulate CD4^+^ T cell responses (43), we examined whether APC-MKs were linked to Treg responses during the postpeak acute-to-early recovery stage of infection. At day 4 after infection, both Treg abundance and the CD25^hi^ fraction within CD4^+^ Foxp3^+^ Tregs were significantly increased, and both parameters showed strong positive correlations with lung MHCII^+^ MK abundance (Figure 6, B–E, and Supplemental Figure 6A). To determine whether this association reflected a broad T cell response or was preferentially related to Tregs, we further analyzed additional T cell subsets. IFN-γ^+^ T cells, representing a Th1-associated population, were reduced at day 4 after infection and tended to negatively correlate with MHCII^+^ MK abundance (Supplemental Figure 6B). IL-4^+^ T cells, representing a Th2-associated population, showed only modest changes and did not display a significant positive correlation with MHCII^+^ MKs (Supplemental Figure 6C). CD8^+^ T cells were increased during this time window and showed a positive correlation with MHCII^+^ MKs (Supplemental Figure 6D). Among these T cell readouts, Treg abundance showed a stronger positive correlation with MHCII^+^ MKs than CD8^+^ T cells, supporting our subsequent focus on the APC-MK–Treg axis.

To functionally examine the APC-MK–Treg axis, we isolated MHCII^–^ and MHCII^+^ MKs from the lung on day 4 after infection and cocultured them with CD3^+^ T cells for 3 days (Figure 6F). Compared with MHCII^–^ MKs, MHCII^+^ MKs significantly increased the frequency of CD4^+^ Foxp3^+^ Tregs, the CD25^hi^ fraction within Tregs and CD25^hi^ Tregs among CD4^+^ T cells (Figure 6G). In contrast, MHCII^+^ MKs did not significantly increase IFN-γ^+^ or IL-4^+^ CD4^+^ T cell fractions, nor CD8^+^ CD122^+^ T cells, which were analyzed as a CD8^+^ Treg-associated readout (69, 70), but increased CD8^+^ T cells (Supplemental Figure 6, E–I). These in vitro findings show that MHCII^+^ MKs enhance Treg-related readouts and CD8^+^ T cells without significantly increasing Th1- or Th2-associated CD4^+^ T cell fractions, nor CD8^+^ CD122^+^ T cells. Given the established role of Tregs in limiting excessive inflammation and supporting tissue repair during pneumonia recovery, we next assessed the in vivo relevance of the APC-MK–Treg axis during the postpeak acute-to-early recovery stage.

To assess the in vivo relevance of this axis, we applied the oropharyngeal DT-based PF4^Cre^-iDTR strategy during the postpeak acute-to-early recovery stage of infection (Figure 7A). At day 4 after infection, DT treatment efficiently reduced total lung MKs and markedly decreased the APC-MK population (Figure 7B). Mac-MKs were also significantly reduced, whereas neu-MKs showed a downward trend but without statistical significance (Supplemental Figure 7, A and B). Analysis of infected lungs revealed that MK-depleted mice exhibited a pronounced reduction in both the frequency and absolute number of Tregs on day 4 after infection (Figure 7C). The CD25^hi^ fraction within CD4^+^ Foxp3^+^ Tregs and the number of CD4^+^ Foxp3^+^ CD25^hi^ Tregs were also significantly decreased (Figure 7D). Consistently, the frequency of CD4^+^ Foxp3^+^ CD25^hi^ Tregs among total CD4^+^ T cells was reduced (Figure 7E). Correlation analysis further demonstrated a strong positive association between MHCII^+^ MK abundance and both total Treg abundance and CD25^hi^ Treg readouts (Figure 7F). Functionally, lung MK depletion, which markedly reduced APC-MKs, was associated with aggravated histological inflammation, increased pulmonary neutrophil accumulation, and higher bacterial burden at day 4 after infection (Figure 7, G and H). Together with the reduced Treg-related responses, these findings support the involvement of APC-MKs in pulmonary inflammatory control during the postpeak acute-to-early recovery stage.

To determine whether impacted Treg responses contributed to the exaggerated inflammatory phenotype after MK depletion, we transplanted Tregs in DT-treated PF4^Cre^-iDTR mice during infection (Supplemental Figure 7C). Treg administration successfully increased Treg number in DT-treated mice (Figure 7I). Compared with DT treatment alone, Treg transfer markedly alleviated histological lung inflammation and reduced pulmonary neutrophil accumulation (Figure 7J). These findings support a functional role for Tregs in controlling inflammation following MK depletion and provide in vivo evidence that the APC-MK–Treg axis plays an important role in promoting inflammatory control during recovery from bacterial pneumonia.

In summary, these findings reveal a temporally coordinated immune program among lung MK subtypes during bacterial pneumonia. Early infection preferentially induces mac-MKs and neu-MKs, which display phagocytic-like and bactericidal potential and contribute to early pulmonary inflammation. Subsequently, the APC-MK–Treg axis promotes inflammatory control. Together, these results highlight the dynamic, stage-specific roles of distinct lung immune MK subtypes, although the underlying mechanisms require further investigation.

## Discussion

In this study, we identified, 3 megakaryocyte (MK) subtypes with distinct immune functions — mac-MKs, neu-MKs, and APC-MKs—through single-cell transcriptomic analysis of both mouse and human samples. These subtypes are broadly distributed across multiple tissues and exhibit pronounced organ-specific distribution patterns, as well as temporal dynamics during development. In a bacterial pneumonia model, mac-MKs and neu-MKs were preferentially induced during the early acute inflammatory stage and showed phagocytic-like and bactericidal potential, while lung MK depletion attenuated early pulmonary inflammation. During the postpeak acute-to-early recovery stage, APC-MKs were associated with a Treg-linked regulatory program that supported the control of pulmonary inflammation. Importantly, our study reveals a previously unrecognized MK-Treg regulatory axis, representing a distinct hematopoietic-immune interaction mechanism. This work not only provides the cellular and functional evidence of MK immune heterogeneity — demonstrating that distinct immune functions are mediated by specific MK subtypes — but also extends our understanding of how nonclassical immune cells contribute to host defense and immune regulation, establishing a foundational framework for exploring MK functions in physiological and pathological contexts.

Recent studies have increasingly highlighted the role of MKs in immune regulation. It is now well established that MKs can express multiple immune-related molecules (43) and are actively involved in pathogen phagocytosis, inflammation modulation, and antigen presentation (29, 48, 49, 51, 52). However, although recent evidence suggested that MK immune functions may be mediated by specific subsets (27–29, 32, 34, 35), MKs with immune characteristics have largely been regarded as a functionally uniform population. The existence and precise features of specialized immune subtypes have not been systematically characterized, limiting the accurate dissection of MK-mediated immune mechanisms. To address this question, our study employed single-cell transcriptomics to systematically reveal MK immune heterogeneity at the molecular level. We found that immune MKs across multiple tissues do not form a single homogeneous subpopulation but comprise three immune-related subtypes: mac-MKs, with phagocytic-like features; neu-MKs, enriched for inflammatory and bactericidal programs; and APC-MKs, with antigen-presentation–related features. Each subtype expressed subtype-enriched markers and effector genes and showed distinct transcriptional regulatory programs that integrated megakaryocytic and immune-related pathways. By identifying subtype-enriched surface markers, we not only confirmed the immune heterogeneity of MKs but also established technical approaches for their precise isolation, providing essential tools for downstream studies. Crucially, we validated the presence of similar immune heterogeneity in human samples, indicating cross-species conservation. At single-cell resolution, these findings enable precise identification and classification of MK subtypes with distinct immune functions, laying a foundation for dissecting their specific roles within complex immune microenvironments and deepening our understanding of MK immune complexity.

We further reveal that MK immune heterogeneity is both organ-specific and developmentally regulated. The 3 immune MK subtypes in adult mouse bone marrow are approximately balanced, whereas in the spleen and lung, APC-MKs are disproportionately abundant relative to the other 2 subtypes. This distribution pattern suggests that MK immune function might be highly specialized according to the physiological and immunological demands of each organ and highlights the role of the tissue microenvironment in shaping subtype composition. Across developmental stages, overall MK immune characteristics strengthen with age, but each immune subtype exhibits distinct temporal dynamics. For example, in the lung, mac-MKs appear during embryogenesis and rapidly expand postnatally, reaching peak abundance in adulthood, whereas neu-MKs and APC-MKs remain scarce during embryonic and neonatal stages, increasing significantly only in adulthood. Notably, the pronounced enhancement of antigen-presenting function in adult lung MKs aligns with prior observations that adult lungs exhibit more efficient immune responses than neonatal lungs, supporting the robustness of these developmental trends (71). Functionally, the early emergence of mac-MKs in the embryonic stage may reflect their role in baseline immune surveillance in a relatively sterile environment, clearing maternal-derived pathogens to provide initial immune protection for the developing embryo. The postnatal upregulation of neu-MKs and APC-MKs indicates that MK immune specialization not only intensifies with development but also diversifies functionally. Similar developmental patterns were observed in human fetal liver and adult bone marrow. Collectively, these findings demonstrate that MK immune heterogeneity might follow strict spatiotemporal developmental rules, with different subtypes participating sequentially and coordinately in constructing the hierarchical architecture of the immune system. This provides critical experimental evidence for understanding the maturation of MK immune functions and suggests that distinct immune subtypes collectively contribute to immune system organization and functional refinement through their temporal and spatial distribution.

We also investigated the dynamic behavior of MK immune subtypes during host immune responses. Mac-MKs and neu-MKs were preferentially induced during the early acute inflammatory stage and displayed phagocytic-like potential. Notably, lung MK reduction attenuated pulmonary inflammation and reduced neutrophil accumulation. This finding suggests that early infection-responsive MK subtypes should not be interpreted simply as protective bacterial-clearance cells. Rather, mac-MKs and neu-MKs may possess inflammatory-amplifying functions. In this acute pulmonary infection setting, the net in vivo effect of the lung MK compartment appears to be the enhancement of local inflammatory responses. By contrast, APC-MKs became more prominent during the postpeak acute-to-early recovery stage and were linked to Treg-related inflammatory control. Together, these findings provide the clear demonstration of temporally regulated activation and functional specialization among MK immune subtypes during pneumonia, with the 3 subtypes acting cooperatively to form an integrated immune defense network. These results provide direct functional evidence that MKs achieve multifaceted immune roles through coordinated actions of distinct specialized subtypes rather than as a uniform population, offering a distinct conceptual framework for understanding MK functional diversity in immune defense and inflammation regulation.

Accumulating evidence has shown that MKs can activate CD4^+^ and CD8^+^ T cells via antigen presentation, contributing to adaptive immune responses. However, whether MKs also modulate Tregs — key mediators of immune homeostasis — remained unclear. Our study demonstrates that MKs can regulate Treg function, and that this effect is mediated by APC-MKs rather than the MK population as a whole. Using an in vivo pneumonia model, we further validated the physiological relevance of this regulatory axis. These findings expand our understanding of MK-mediated T cell modulation and reveal a Treg-linked mechanism by which immune MK subtypes contribute to pulmonary inflammatory control.

In summary, this study demonstrates substantial immune-related heterogeneity in MKs. These findings expand our understanding of MK biological complexity and provide a framework for studying immune-related heterogeneity in other nonclassical immune-regulatory cells (72–76). Nevertheless, several key questions remain: First, can MKs with distinct immune properties produce functionally specialized platelets, thereby transmitting their immune characteristics to its progeny? Second, what are the origins and differentiation pathways of these specialized immune MKs — do they arise from a common progenitor that diverges, or from independent developmental lineages (77, 78)? Third, how do these subtypes interact with classical immune cells (such as macrophages, neutrophils, and dendritic cells) in terms of development, function, and regulation? Continued investigation of these questions will further illuminate the role of MKs within the immune system and their physiological and pathological significance.

## Methods

### Sex as a biological variable.

Our study examined male and female human participants and animals, and similar findings are reported for both sexes.

### Mice.

PF4^Cre^-iDTR mice were used for MK depletion studies. Flt3^Cre^; Rosa26^tdTomato^ mice were obtained from Shanghai Biomodel Organism Co., Ltd. C57BL/6 mice were purchased from the animal facility of the State Key Laboratory of Experimental Hematology. Adult mice used in this study were 8- to 12-week-old healthy mice.

### Single-cell suspension for scRNA-seq, flow cytometry, ImageStream flow cytometry, and cell culture.

For MK isolation from mouse spleen and lung, tissues were enzymatically digested using DNase I (Sigma, 9003-98-9) and collagenase (Sigma, C2674) at 37°C for 30 minutes to obtain single-cell suspensions. All cell suspensions were treated with ACK lysis buffer and filtered through a 100 μm cell strainer. For both human and mouse bone marrow, ACK lysis buffer (Solarbio, R1010) was added to remove red blood cells. Human bone marrow cells were isolated and enriched by bovine serum albumin (BSA, Solarbio, A8020) gradient centrifugation.

### Single-cell RNA-seq.

MKs labeled with antibodies were sorted using a FACS AriaIII flow cytometer (BD Biosciences). Individual MKs were transferred into lysis buffer under a microscope using micromanipulation. Single-cell RNA-Seq libraries were prepared using the STRT-seq (28) method and sequenced on the Illumina NovaSeq6000 platform (Barry Genomics).

### Sequencing data processing.

Raw sequencing data were processed using Cutadapt software to remove reads containing poly-A tails, TSO and other sequencing element sequences, as well as low-quality sequences, yielding clean reads. These reads were aligned to the reference genome ENSEMBL GRCm38.93 using STAR (79) software. Each cell was matched to a unique barcode sequence to generate a gene expression matrix. Low-quality cells were removed if the number of detected genes per cell was < 500, the UMI (unique molecular identifiers) count was < 1000, or mitochondrial gene expression exceeded 3 standard deviations above the mean. After quality control, 368 adult bone marrow MKs, 247 adult spleen MKs, and 971 MKs from lungs at different developmental stages were obtained. To eliminate batch effects between sequencing runs, Seurat (v4.1.1) functions FindIntegrationAnchors and IntegrateData were used to integrate samples based on the top 2000 highly variable genes with default parameters. To further analyze heterogeneity, the Elbow method was used to select relevant PCs for dimensionality reduction. Clustering was performed using the top 20 PCs with the FindClusters function and resolution = 0.8. Five MK subpopulations were identified and annotated based on marker genes highly expressed in each cluster. The FindAllMarkers function in Seurat (80) (with Wilcoxon rank-sum test, min.pct = 0.25) was used to identify feature genes for each subpopulation, *P* values were adjusted for multiple testing using the Benjamini-Hochberg method, and genes with an adjusted *P* value < 0.05 were considered statistically significant. For P7 MKs, we used SingleR (81) (1.6.1) to map 8W immune annotations into P7 immune MKs. For human MKs, we integrated immune MK subpopulations from previously published data and further divided them into three immune MK subtypes.

### Differential gene analysis.

GO functional enrichment analysis of subpopulation-specific DEGs was performed using the online tool g:Profiler (https://biit.cs.ut.ee/gprofiler/gost), selecting representative GO terms with adjusted *P* < 0.05. For gene set enrichment analysis (GSEA), DEGs were ranked by log2FC, *P*-value, or t-statistics to create a list from most upregulated to most downregulated. Gene sets were obtained from the Gene Ontology database (http://geneontology.org/), and enrichment scores (ES) were calculated using the R package fgsea (v1.18.0). Enrichment plots were generated to visualize gene set distribution in the ranked gene list. Additionally, public gene sets from the Gene Ontology database were used, and Seurat’s AddModuleScore function scored each cell based on gene expression. The ggpubr package was used to compare scores between groups using Wilcoxon tests.

### Principal component analysis.

Average normalized expression values of MKs at different time points were exported using Seurat’s AverageExpression function. Genes with normalized expression > 0 were selected for PCA using the prcomp function, and results were visualized using ggplot2 (v3.5.1).

### Transcription factor regulatory analysis.

The pySCENIC pipeline was used to infer transcriptional regulons (transcription factors and their target genes) and their activity in different immune MKs. First, co-expression modules were inferred. Second, using databases (mouse: mm9-tss-centered-10kb-7species.mc9nr.feather), TF activities in single cells were quantified based on genome-wide gene expression rankings. Finally, a cellular regulon enrichment matrix (AUCell) was constructed and visualized with pheatmap (v1.0.13).

### Flow cytometry and Imaging flow cytometry for immune MK subtypes.

Single-cell suspensions were prepared as previously described. For each sample, 1–5 × 10^6^ cells were stained with Fc receptor blocker (Biolegend, 101320) and a panel of fluorophore-conjugated antibodies at 4°C in the dark for 30 minutes. The antibody panel for mouse samples included a lineage antibody cocktail consisting of PE/Cyanine7 anti-mouse Ter-119 antibody (Biolegend, 116222), PE/Cyanine7 anti-mouse/human CD11b antibody (Biolegend, 101216), PE/Cyanine7 anti-mouse CD3 antibody (Biolegend, 100220), and PE/Cyanine7 anti-mouse/human CD45R/B220 antibody (Biolegend, 103222); MK markers, including FITC anti-mouse CD41 antibody (Biolegend, 133904) and antibody for In Vivo Mouse Platelet Labeling (CD42C, Emfret, X649), as well as imm^+^ MK markers, including PE anti-mouse F4/80 antibody (Biolegend, 123110), PE anti-mouse Ly-6G (BD bioscience, 551461), and APC/Cyanine7 anti-mouse I-A/I-E antibody (Biolegend, 107628). While the human panel included FITC mouse anti-human CD41a antibody (BD bioscience, 555466), APC mouse anti-human CD42b antibody (BD bioscience, 551061), as well as imm^+^ MK markers, including PE/Cyanine7 anti-human CD163 antibody (Biolegend, 326514), PE anti-human CD177 antibody (Biolegend, 315806) and APC/Cyanine7 anti-human HLA-DR antibody (Biolegend, 307618). Absolute counting beads were added to quantify cell count, and 7AAD (Biolegend, 420403 and BD bioscience, 559925), Zombie violet (Biolegend, 42311) or DAPI (Solarbio, C0060) dye was added to exclude dead cells. For the gating strategy, cells were first selected based on their FSC/SSC profiles, followed by the exclusion of doublets, dead cells, and lineage-high cells. Within the CD41^+^ CD42c^+^ MK population, immune MK subtypes were further characterized based on the expression of immune lineage-associated markers: F4/80^+^ Mac-MKs, Ly6G^+^ Neu-MKs, and MHCII^+^ APC-MKs.

Mouse samples used for imaging flow cytometry were prepared from adult bone marrow, spleen, and lung single-cell suspensions. The antibody panel included CD41, CD42c, F4/80, Ly6G, MHCII, and DAPI. Samples were acquired using an Amnis ImageStreamX Mark II imaging flow cytometer (Cytek Biosciences) and analyzed using IDEAS software.

### Confocal microscopy.

Mouse bone, spleen, and lung tissues were fixed in 4% paraformaldehyde (PFA, Solarbio, P1110) for 24 hours. Bone tissues were decalcified in 0.5 M EDTA (Solarbio, E1170) for 2 days and dehydrated in 30% sucrose (Solarbio, S8271) for 24 hours. Tissues were then cryosectioned at 20 μm thickness. Sections were stained with antibodies against FITC anti-mouse CD41 (BD Bioscience, 553848, 1:200), APC anti-mouse F4/80 Antibody (Biolegend, 123116, 1:150), APC anti-mouse Ly-6G Antibody (Biolegend, 127614, 1:150), APC anti-mouse I-A/I-E Antibody (Biolegend, 107614, 1:150), and DAPI (Solarbio, C0060, 1:200). Confocal images were captured using a spinning disk confocal microscope (Dragonfly, Andor) and analyzed with Imaris 9.0 software (Oxford Instruments).

### Mouse pneumonia model.

C57BL/6 mice received 3 × 10^6^ CFU of freshly cultured *E*. *coli* (strain 19138; American Type Culture Collection) in 50 μL saline into the lungs via tracheal instillation. To evaluate pneumonia progression, mice were analyzed at indicated time points post-infection (days 0, 1, 4, 7, and 10). Bronchoalveolar lavage fluid (BALF) was collected for measurement of inflammatory cytokines, including IL-6 and TNF-α. Following euthanasia, lungs were harvested, and processed into single-cell suspensions for assessment of bacterial burden (*E*. *coli*) and neutrophil infiltration.

### Murine in vivo and human in vitro phagocytosis models.

C57BL/6 mice received 1 × 10^7^ GFP^+^ *E*. *coli* into the lungs via tracheal instillation. At 24 hours post-infection, lung MKs were analyzed by flow cytometry of GFP^+^ bacterial signal.

Human bone marrow cells were enriched by BSA density gradient centrifugation and co-cultured with 1 × 10^7^ GFP^+^ *E*. *coli* for 2 hours in vitro, human bone marrow MKs were analyzed by flow cytometry of GFP^+^ bacterial signal.

### In vitro bacterial killing assay and quantification of bacterial burden in vivo.

Freshly cultured *E*. *coli* (strain 19138; ATCC) was suspended in saline at OD600 = 0.20 and opsonized in 10% mouse serum at 37°C with shaking for 30 minutes. Purified 1 × 10^5^ lung neu-MKs or non-neu-MKs cells were incubated with 1 × 10^5^ *E*. *coli* for 2 hours. After incubation, cells were plated on LB agar plates. CFUs were counted after overnight incubation at 37°C. Bacterial suspensions without cells served as controls. The reduction in CFUs indicated bacterial killing ability.

To assess bacterial burden in vivo, mice were euthanized at indicated time points (0, 1-, 4-, 7-, and 10-days post-infection). The right lung lobes were harvested, homogenized, filtered through a cell strainer, and serially diluted. Diluted lung homogenates were plated on LB agar plates, and bacterial colony-forming units (CFUs) were quantified after overnight incubation at 37°C.

### Measurement of Tregs and other T cell subsets.

For in vivo studies, single-cell suspensions of mouse lungs were prepared as described above. For in vitro co-culture studies, T cells after 3 days of culture were collected, centrifuged, and resuspended. Viable cells were stained for surface markers including FITC anti-mouse CD3 antibody (Biolegend, 100204), APC anti-mouse CD4 antibody (Biolegend, 100412), and APC/Cyanine7 anti-mouse CD25 antibody (Biolegend, 101918), followed by intracellular PE-Foxp3 Monoclonal Antibody (eBioscience, 12-5773-82) staining using a Foxp3 staining kit (eBioscience, 00-5523-00). CD4^+^ Foxp3^+^ cells were defined as Tregs, and the CD25^hi^ fraction within CD4^+^ Foxp3^+^ Tregs was analyzed as an activation-associated Treg phenotype. The proportion and number of Tregs as well as active Tregs were analyzed by flow cytometry.

Other T cell subsets, including Th1, Th2, CD8^+^ T cells, and CD8^+^ Tregs. Single-cell suspensions were prepared from lung tissues, and stimulated with Cell Activation Cocktail in the presence of Protein Transport Inhibitor Cocktail for 6 h to induce intracellular cytokine accumulation. After stimulation, Th1 and Th2 cells were stained with a fixable viability dye and surface antibodies against CD3 and CD4. Cells were then fixed and permeabilized using an intracellular staining buffer, followed by staining with PE anti-mouse IFN-r antibody (Biolegend, 163504) and PE/Cyanine7 anti-mouse IL-4 antibody (Biolegend, 504118). Th1 and Th2 cells were identified by flow cytometry as CD3^+^ CD4^+^ IFN-γ^+^ and CD3^+^ CD4^+^ IL-4^+^ T cells. CD8^+^ T cells were identified by expression of PE/Cyanine7 anti-mouse CD8a antibody (Biolegend, 100722), whereas CD8^+^ Tregs were identified by expression of PE anti-mouse CD122 (IL-2Rb) antibody (Biolegend, 105906) within the CD8^+^ T cell population.

### In vitro co-culture assay of APC-MKs with T cells.

CD3^+^ T cells were isolated from single-cell suspensions of *E*. *coli*-infected mice spleen using the CD3^+^ T Cell Isolation Kit (Stemcell, 19851). A total of 5 × 10^4^ T cells were co-cultured with either MHCII^+^ MKs or MHCII^–^ MKs (sorted from *E*. *coli*-infected mice lung single-cell suspensions) at a 1:1 ratio, in the presence of recombinant murine IL-2 (PeproTech, 212-12-20UG), CD3 monoclonal antibody (Invitrogen, 16-0031-86) and CD28 monoclonal antibody (Invitrogen, 16-0281-86) for 3 days. After co-cultivation, the proportion of T cell subsets were analyzed by flow cytometry.

### Oropharyngeal DT-mediated lung MK depletion model.

For local reduction of lung MKs, DT (Sigma, D0564-1MG; 0.5 ng/g body weight) or saline was delivered into the airways of PF4^Cre^-iDTR mice by oropharyngeal administration in a volume of 50 μL. To evaluate whether DT administration affects pulmonary immune cells, C57BL/6 mice received oropharyngeal administration of DT or saline on days 0 and 2. Lung immune cell composition was assessed on day 4: Ly6G^+^ neutrophils, F4/80^+^ macrophages, CD3^+^ T cells, and CD19^+^ B cells (PE/Cyanine7 anti-mouse CD19 antibody, Biolegend, 152418). To verify that DT selectively depletes lung MKs without affecting MKs in other organs, PF4^Cre^-iDTR mice were treated with DT or saline on Days 0 and 2 using the same strategy. The proportions of MKs in the lung, bone marrow, and spleen were analyzed on Day 4.

For early-phase MK depletion in pneumonia, mice were administered DT or saline on days −3 and −1, followed by *E*. *coli* challenge on day 0. Lungs were collected and analyzed at day 1 post-infection. For post-peak acute-to-early recovery experiments, mice were administered DT or saline on days 0 and 2, challenged with *E*. *coli* on day 0, and lungs were collected for analysis at day 4 post-infection.

### Adoptive Tregs transplantation model.

Single-cell suspensions were prepared from mouse spleens by dissociation followed by filtration. Tregs were enriched using the Mouse CD4^+^ CD25^+^ Regulatory T Cell Isolation Kit II (Stemcell, #18783) according to the manufacturer’s instructions. Using the established lung MK depletion model, mice received DT treatment on day 0, followed by adoptive transplantation of 1 × 10^6^ purified Tregs on day 2. On day 4, lungs were harvested to quantify pulmonary Tregs and evaluate the extent of inflammatory infiltration during pneumonia.

### Resources.

Details of materials used in this paper, including antibodies, chemicals, commercial assays, software, and algorithms, are in the Key Resources Table (Supplemental Table 4).

### Statistics.

For single-cell analysis, differences between 2 groups were assessed using the nonparametric Wilcoxon test in R. *P* values were determined using 2-sided Wilcoxon rank-sum tests and adjusted for multiple comparisons using the Benjamini-Hochberg method. For biological experiments, statistical analyses were performed using GraphPad Prism 10.0 (GraphPad Software, Inc.). Comparisons between 2 groups were conducted using 2-tailed unpaired *t* tests. Comparisons among multiple groups were performed using 1-way ANOVA followed by Tukey’s multiple-comparison test. Linear regression analysis was performed to evaluate the correlation between 2 groups. The strength of the association was assessed by the coefficient of determination (R^2^), and statistical significance was determined by the corresponding *P* value. Data are presented as mean ± SEM. *P* values < 0.05 were considered significant.

### Study approval.

All animal experiments were approved by the Animal Welfare and Ethics Committee of the Institute of Hematology & Blood Diseases Hospital, Chinese Academy of Medical Sciences & Peking Union Medical College (Approval number: IHCAMS-DWLL-NSFC2025157-1).

Human bone marrow samples were obtained with informed consent and approved by the Ethics Committee of the Institute of Hematology, Chinese Academy of Medical Sciences (Approval number: NSFC2021003-EC-2) from healthy donors (*n* = 10; median age, 39 years; range, 23–52 years; 3 female and 7 male).

### Data availability.

The embryonic lung MK single-cell RNA-seq data generated in this study have been deposited in the National Genomics Data Center under accession number OMIX019453.

The human MK single-cell RNA-seq datasets used for the analysis in this study are publicly available in the Gene Expression Omnibus (GEO) under accession number GSE144024 and in the National Omics Data Encyclopedia (NODE) under accession number OEP001150. The mouse bone marrow, spleen, and adult lung MK datasets are available in the OMIX database under accession number OMIX015596. The immune-cell dataset is available in GEO under accession number GSE116530.

Values for all data points in graphs are reported in the Supporting Data Values file.

## Manuscript information

### Authorship contributions.

HH, YM, YC, and XC contributed equally to this work. JZ, HW, and CL coordinated and designed the project. HH, XC, and MX provided the single-cell RNA-seq data of mouse MKs. YM and ML did the transcriptome analysis. HH, YC, XC, TC, ZH, and SG performed experiments and data acquisition. DF, YC, JZ, and NZ provided new methods. PS, WZ, and FW provided new insights. JZ, HW, CL, and FW wrote, reviewed and edited the manuscript.

### AI disclosure.

During the preparation of this work, the authors used ChatGPT 5.6 (OpenAI; licensed to FW) to improve the clarity and quality of the scientific writing. After using this tool, the authors reviewed and edited the content as needed and take full responsibility for the content of the published article. No other artificial intelligence tools were used in the preparation of this manuscript.

### Conflict of interest.

HW is a founder and shareholder of HaemoCure Inc. CL is a shareholder of HaemoCure Inc. FW is an employee of HaemoCure Inc.

### Funding.

This work was supported by the following:

- Innovative Drug Research and Development National Science and Technology Major Project (2025ZD1803600 to JZ).
- CAMS Innovation Fund for Medical Sciences (CIFMS) (2026-I2M-SXZX14-02 to JZ, 2026-I2M-SXZX17-01 to HW, 2025-12M-XHZY-035 to JZ, 2025-12M-XHXX-133 to CL).
- National Natural Science Foundation of China (82125003 to JZ, 82430009 to JZ, 32271161 to HW, 32471167 to HW and 32571301 to CL).
- Science and Technology Projects of Xizang Autonomous Region, China (XZ202502ZY0031 to HW).
- Natural Science Foundation of Tianjin (24JCJQJC00110 to HW).

## Supplementary Material

Supplemental data

Supplemental tables 1-3

Supporting data values

## Figures and Tables

**Figure 1:**
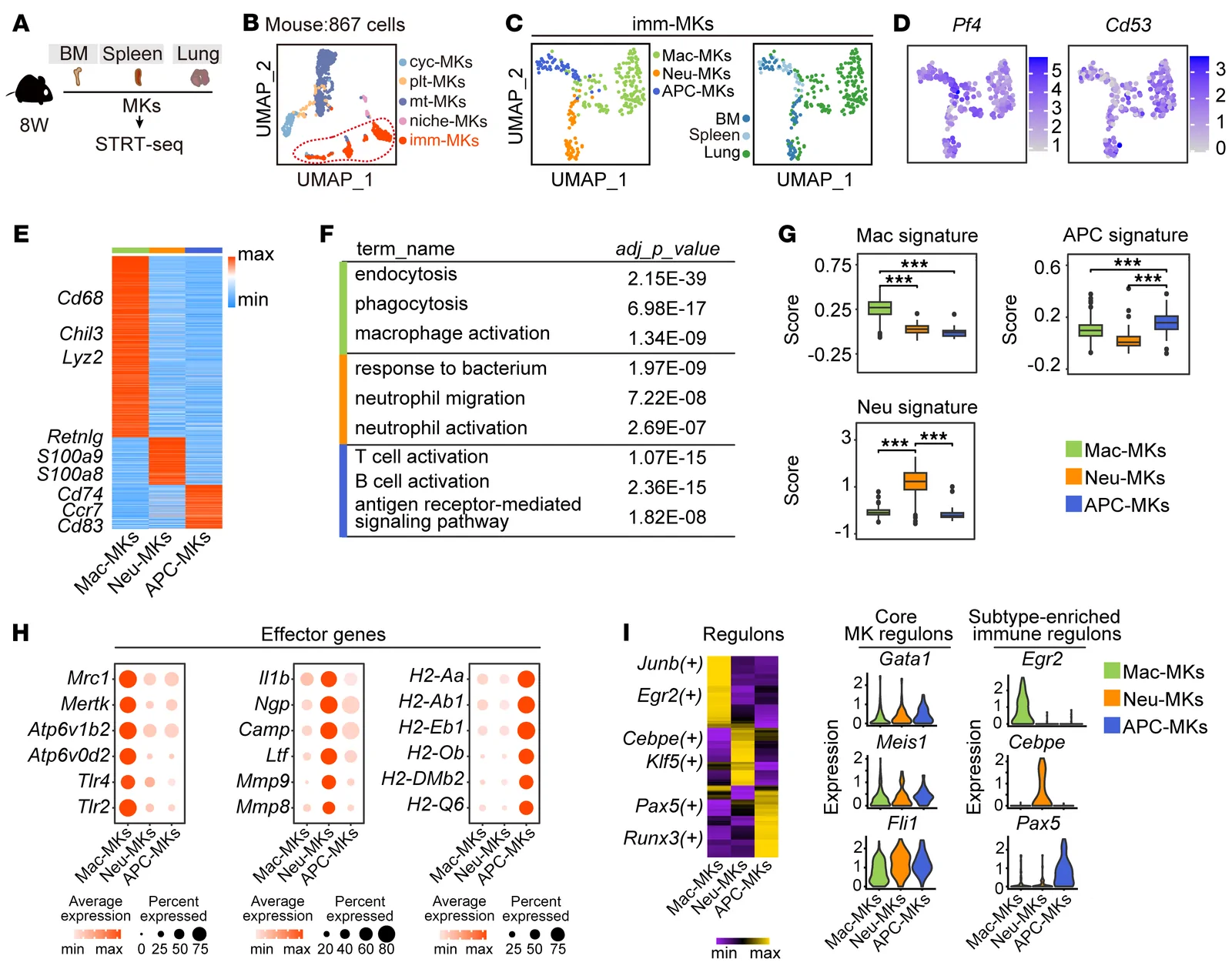
Mouse megakaryocytes exhibit significant immune heterogeneity. (**A**) Schematic of mouse multiorgan MK isolation for single-cell RNA-seq. (**B**) UMAP plot showing distribution of the MK subpopulations across multiple organs. (**C**) UMAP plots showing distribution of immune MK subtypes (left) and their organ origins (right). (**D**) Featureplots showing expression of MK (left) and immune (right) marker genes in immune MKs. (**E**) Heatmap of differentially expressed genes of immune MK subtypes. (**F**) GO enrichment results among immune MK subtypes. (**G**) Boxplots showing immune signature scores of different immune MK subtypes (Supplemental Table 3A). The median is indicated by a horizontal line, and boxes represent the first and third quartiles. (**H**) Dotplots showing expression of immune effector genes across immune MK subtypes. (**I**) Heatmap (left) showing transcriptional regulatory activity across immune MK subtypes; violin plot (right) showing expression of MK and immune-feature transcription factors. Statistical analyses were performed using using 2-sided Wilcoxon rank-sum tests and adjusted for multiple comparisons using the Benjamini-Hochberg method for **E** and **G**; *P* < 0.05 considered significant, ***P < 0.001.

**Figure 2:**
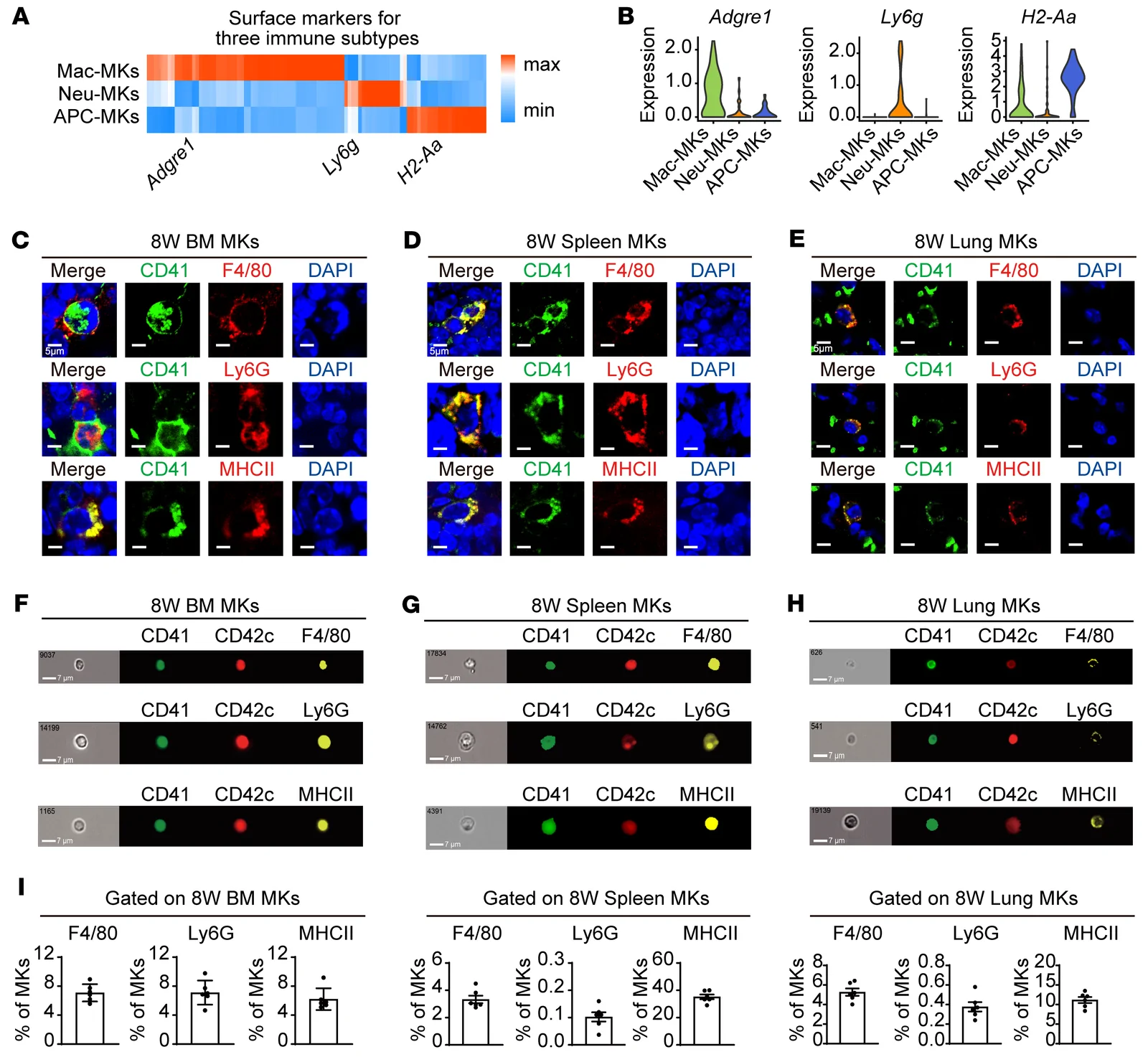
Successful identification of mac-MKs, neu-MKs, and APC-MKs. (**A**) Heatmap displaying surface marker screening for different immune MK subtypes. (**B**) Violin plots showing expression of *Adgre1*, *Ly6g*, and *H2-Aa* across immune MK subtypes. (**C**) Immunofluorescence images showing CD41^+^ MKs with F4/80, Ly6G, or MHCII signals in adult bone marrow. Scale bar: 5 μm. (**D**) Immunofluorescence images showing CD41^+^ MKs with F4/80, Ly6G, or MHCII signals in adult spleen. Scale bar: 5 μm. (**E**) Immunofluorescence images showing CD41^+^ MKs with F4/80, Ly6G, or MHCII signals in adult lung. Scale bar: 5 μm. (**F**) Representative imaging flow cytometry images showing CD41, CD42c, and F4/80, Ly6G, or MHCII signals in CD41^+^ CD42c^+^ MK events from adult bone marrow. Scale bar: 7 μm. (**G**) Representative imaging flow cytometry images showing CD41, CD42c, and F4/80, Ly6G, or MHCII signals in CD41^+^ CD42c^+^ MK events from adult spleen. Scale bar: 7 μm. (**H**) Representative imaging flow cytometry images showing CD41, CD42c, and F4/80, Ly6G, or MHCII signals in CD41^+^ CD42c^+^ MK events from adult lung. Scale bar: 7 μm. (**I**) Flow cytometry showing proportions of immune MKs in adult bone marrow, spleen, and lung (flow-cytometry validation was performed using 8–12-week-old adult mice). Data are presented as mean ± SEM from 3 independent biological experiments for **I**. Representative immunofluorescence images of lung sections from 3 independent experiments, each yielding similar results.

**Figure 3:**
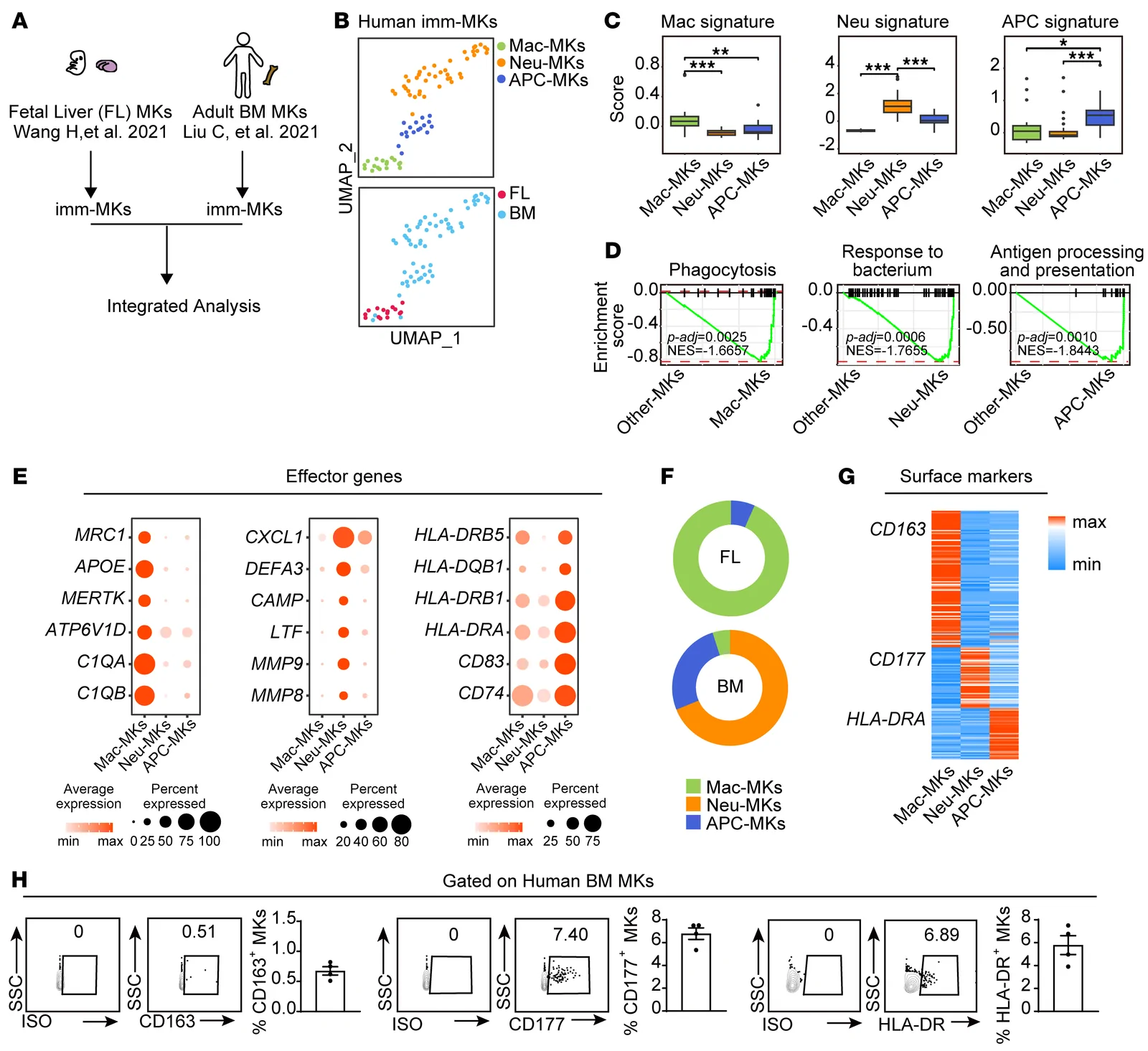
Human megakaryocytes exhibit similar immune heterogeneity. (**A**) Schematic of integrated analysis of fetal liver and adult bone marrow MKs. (**B**) UMAP plots showing the subtypes (top) and organ distribution (bottom) of immune MKs. (**C**) Boxplots showing immune signature scores of different immune MK subtypes (Supplemental Table 3B). The median is indicated by a horizontal line, and boxes represent the first and third quartiles. (**D**) GSEA showing enrichment of functional gene sets in different immune MK subtypes. (**E**) Dotplots showing expression of immune effector genes across immune MK subtypes. (**F**) Donut charts showing distribution of immune MK subtypes across human organs. (**G**) Heatmap showing surface marker screening across immune MK subtypes. (**H**) Flow cytometric analysis of CD163^+^, CD177^+^, and HLA-DR^+^ populations within CD41a^+^ CD42b^+^ human bone marrow MKs. *n* = 4 per group. Data are presented as mean ± SEM from 4 independent biological experiments for **H**; Statistical analyses were performed using 2-sided Wilcoxon rank-sum tests and adjusted for multiple comparisons using the Benjamini-Hochberg method for **C**; *P < 0.05, **P < 0.01, ***P < 0.001.

**Figure 4:**
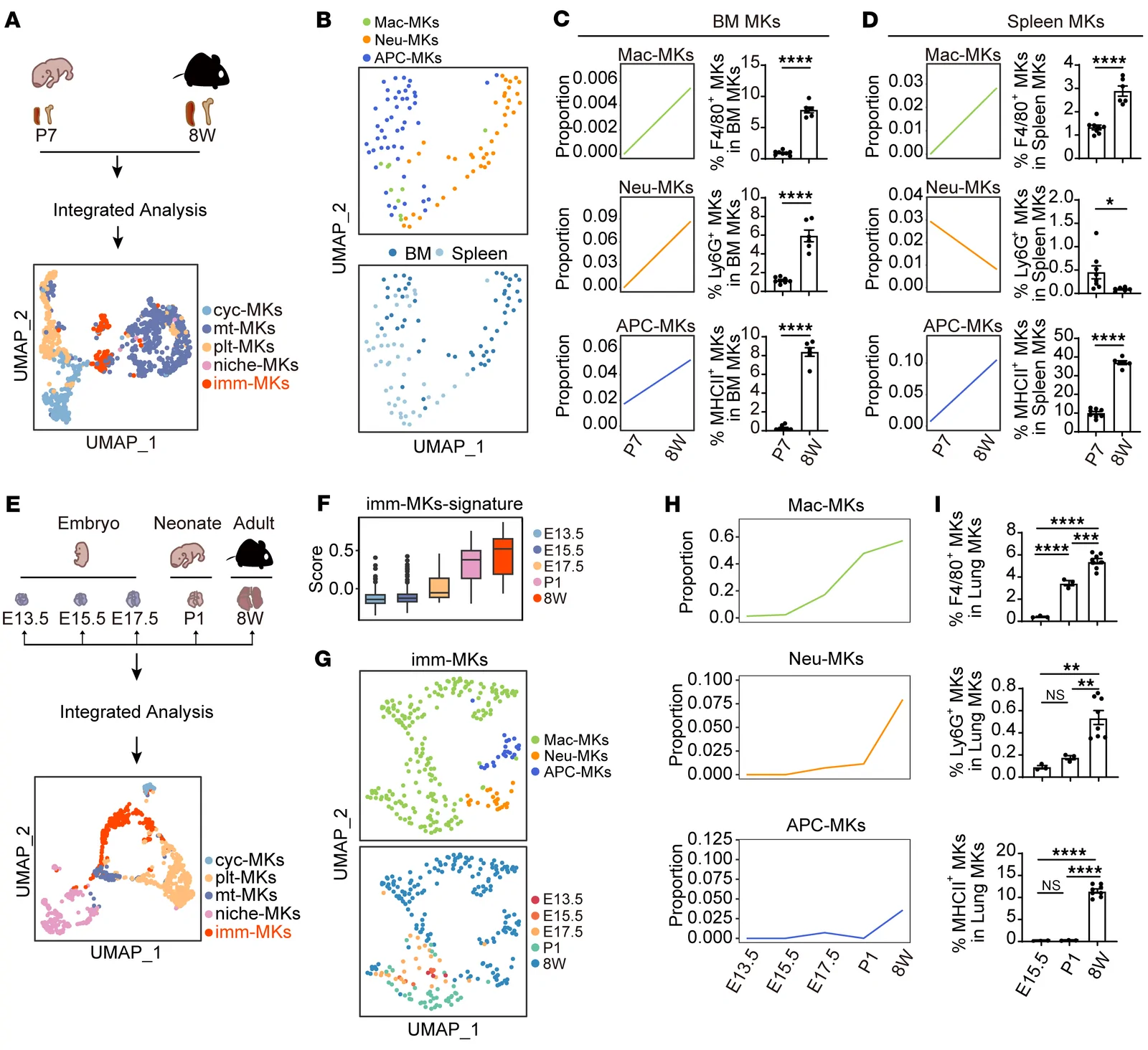
Temporal remodeling of immune megakaryocyte subtypes during development. (**A**) Schematic of integrated transcriptomic analysis of MKs in mouse bone marrow and spleen at different developmental stages (top). UMAP plot showing distribution of MK subpopulations across these 2 organs at different stages (bottom). (**B**) UMAP showing immune MK subtypes (top) and organ distribution (bottom). (**C**) Line charts (left) showing developmental trends (P7 neonatal mice and 8W adult mice) of immune MK subtypes in bone marrow. Bar chart (right) showing flow cytometry data across stages. (**D**) Line charts (left) showing developmental trends (P7 neonatal mice and 8W adult mice) of immune MK subtypes in spleen. Bar chart (right) showing flow cytometry data across stages. (**E**) Schematic of integrated transcriptomic analysis of MKs in mouse lung at different developmental stages (top). UMAP plots highlighting lung immune MK subpopulations (bottom). (**F**) Boxplot showing changes in immune signatures of lung MKs across development. (**G**) UMAP plots showing immune MK subtypes (top) and timepoint distribution (bottom). (**H**) Line charts showing proportions of each immune MK subtype during development. (**I**) Flow cytometry showing proportion changes of immune MK subtypes in lung over development. Data are presented as mean ± SEM. For **C** and **D**, *n* = 8 biological replicates from P7 mice, and 6 biological replicates from 8W mice. For **I**, *n* = 3 biological replicates from E15.5 mice, 3 biological replicates from P1 mice and 8 biological replicates from 8W mice. Statistical analyses were performed using 2-tailed unpaired Student’s *t* test for **C** and **D**, and 1-way ANOVA followed by Tukey’s multiple-comparison test for **I**. *P < 0.05, **P < 0. 01, ***P < 0.001, ****P < 0.0001.

**Figure 5:**
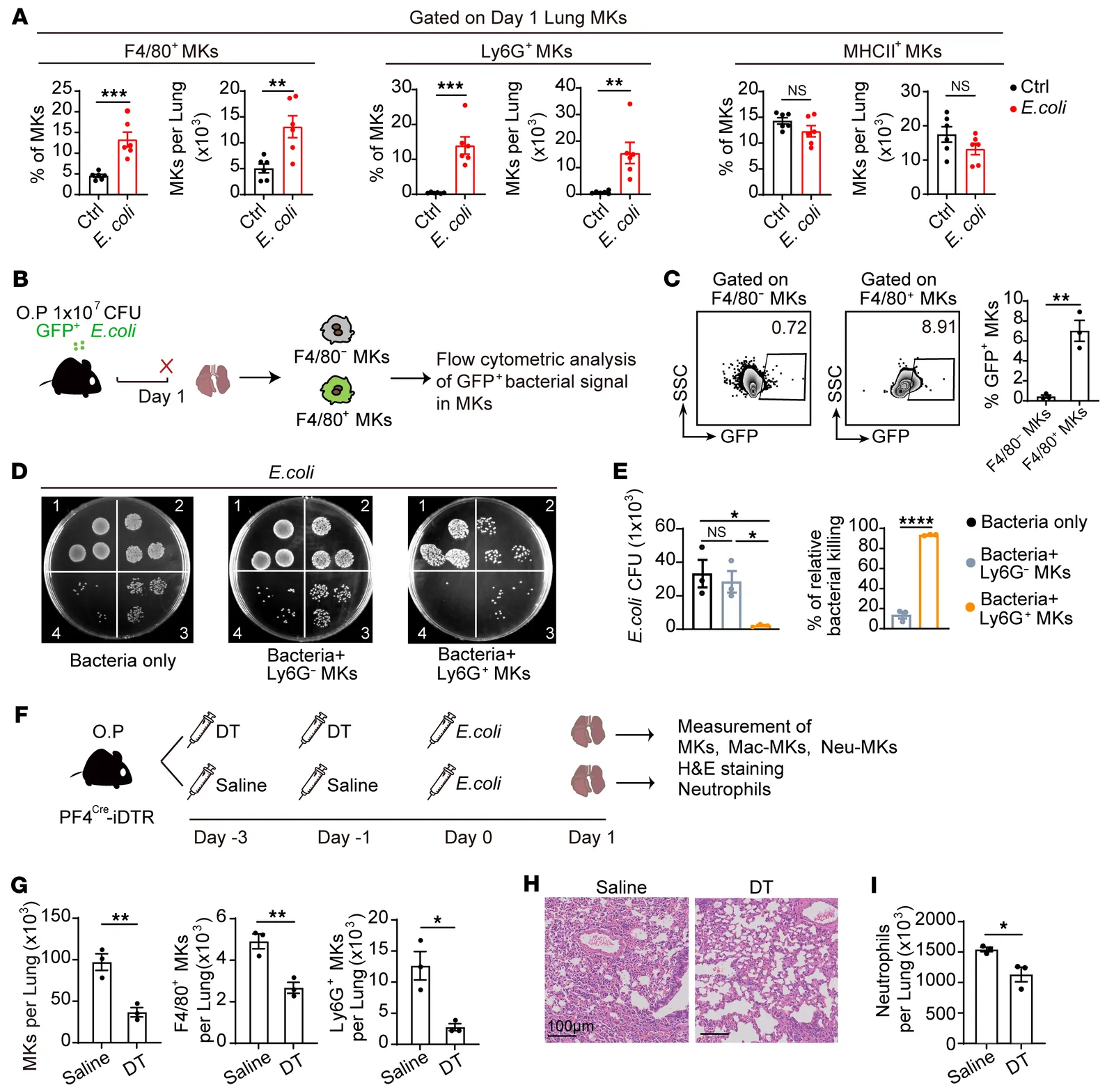
Mac-MKs and neu-MKs contribute to early pulmonary inflammation during bacterial pneumonia. (**A**) Flow cytometry showing early-stage changes in immune MK proportions and numbers in lungs during pneumonia (8–12-week-old age- and sex-matched adult mice). (**B**) Schematic diagram of the in vivo GFP^+^ *E*. *coli* signal-detection assay. (**C**) Flow cytometry analysis of GFP-positive bacterial signals in F4/80^+^ and F4/80^–^ MKs after in vivo GFP^+^ *E*. *coli* infection. Flow cytometry in representative plots (left), statistical results (right). (**D**) Plating assay showing bactericidal capacity of neutrophil-marker–positive versus –negative MKs in coculture (1–4 indicate serial dilutions: undiluted, 10-fold, 100-fold, and 1,000-fold dilution, respectively). (**E**) Residual bacterial count (left) and relative bactericidal rate (right) for neutrophil-marker–positive versus –negative MKs. (**F**) Schematic illustration of the lung MK depletion strategy during the early stage of pneumonia. (**G**) Flow cytometric quantification of total lung MKs, mac-MKs, and neu-MKs in control and DT-treated mice. (**H**) Representative H&E-stained lung sections from control and DT-treated mice. Scale bar: 100 μm. (**I**) Flow cytometric quantification of pulmonary neutrophils in control and DT-treated mice. Data are presented as mean ± SEM from 3 independent biological experiments for **A**, **D**, and **E**, 2 independent biological experiments for **C** and **G**–**I**. Statistical analyses were performed using 2-tailed unpaired Student’s *t* test for **A**, **C**, **E**, **G**, and **I**, and 1-way ANOVA followed by Tukey’s multiple-comparison test for **E**. *P < 0.05, **P < 0. 01, ***P < 0.001, ****P < 0.0001. Representative H&E-stained lung sections from 3 mice per group. Similar results were observed across mice.

**Figure 6:**
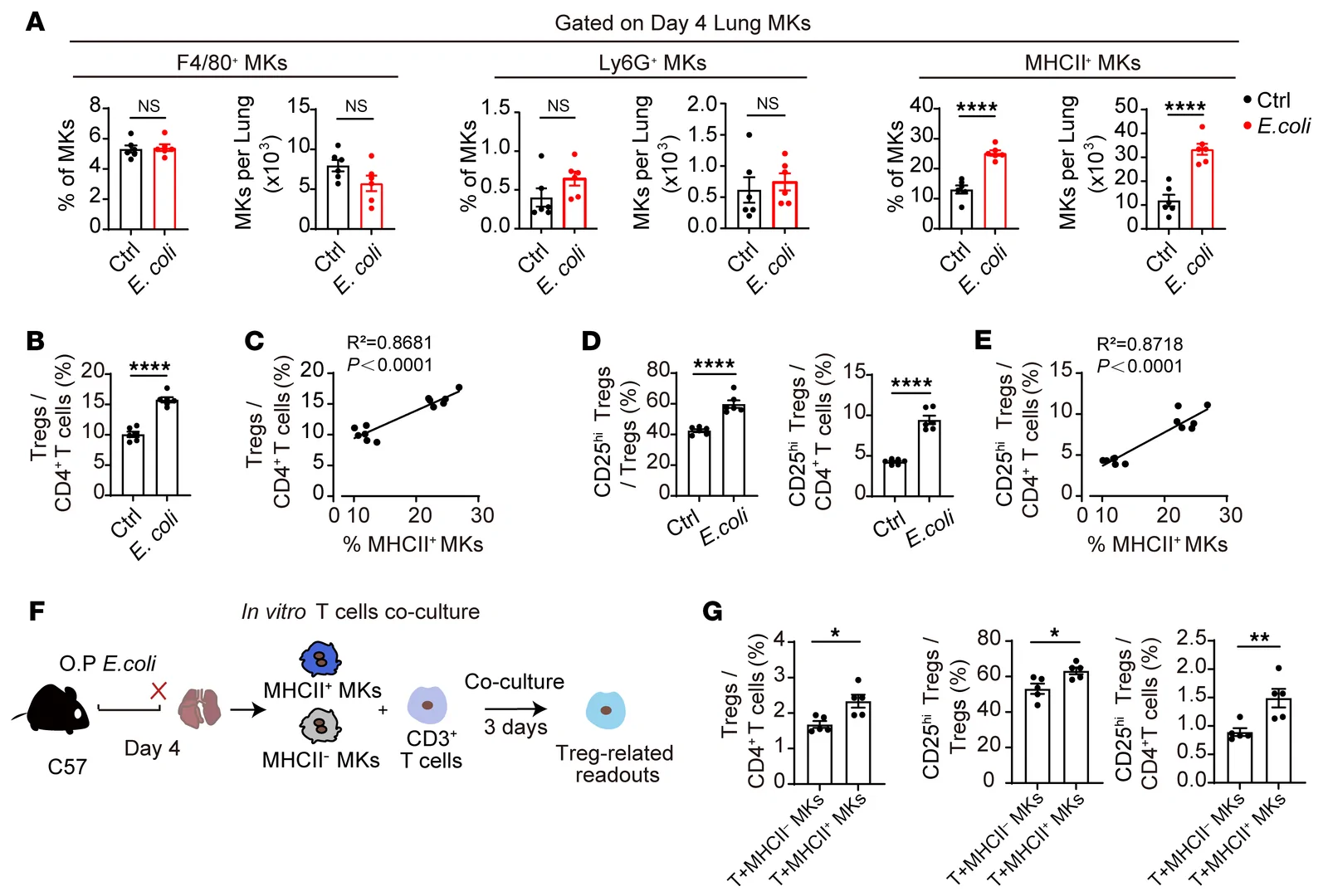
APC-MKs are linked to Treg-associated responses during the postpeak acute-to-early recovery stage of bacterial pneumonia. (**A**) Flow cytometry analysis showing late-stage changes in immune MK proportions and numbers in lungs during pneumonia. (**B**) Flow cytometry analysis of Treg ratios in control versus pneumonia mice. (**C**) Correlation analysis between the proportion of MHCII^+^ MKs and Tregs (R^2^ = 0.8681, *P* < 0.0001) in lungs of pneumonia mice. (**D**) Flow cytometry analysis of the ratios of CD25^hi^ Tregs in Tregs (left) and CD4^+^ T cells (right) in control versus pneumonia mice. (**E**) Correlation analysis between the proportions of MHCII^+^ MKs and CD25^hi^ Tregs in CD4^+^ T cells (R^2^ = 0.8718, *P* < 0.0001) in lungs of pneumonia mice. (**F**) Schematic of the in vitro coculture model of MHCII^+^ MKs and T cells. (**G**) Flow cytometry analysis of Treg ratios in T cells cocultured with MHCII^–^ MKs versus MHCII^+^ MKs. Data are presented as mean ± SEM from 3 independent biological experiments for **A** and **G**, 2 independent biological experiments for **B**–**E**. Statistical analyses were performed using a 2-tailed unpaired Student’s *t* test for **A**, **B**, **D**, and **G**, and linear regression analysis for **C** and **E**. *P < 0.05, **P < 0. 01, ****P < 0.0001.

**Figure 7:**
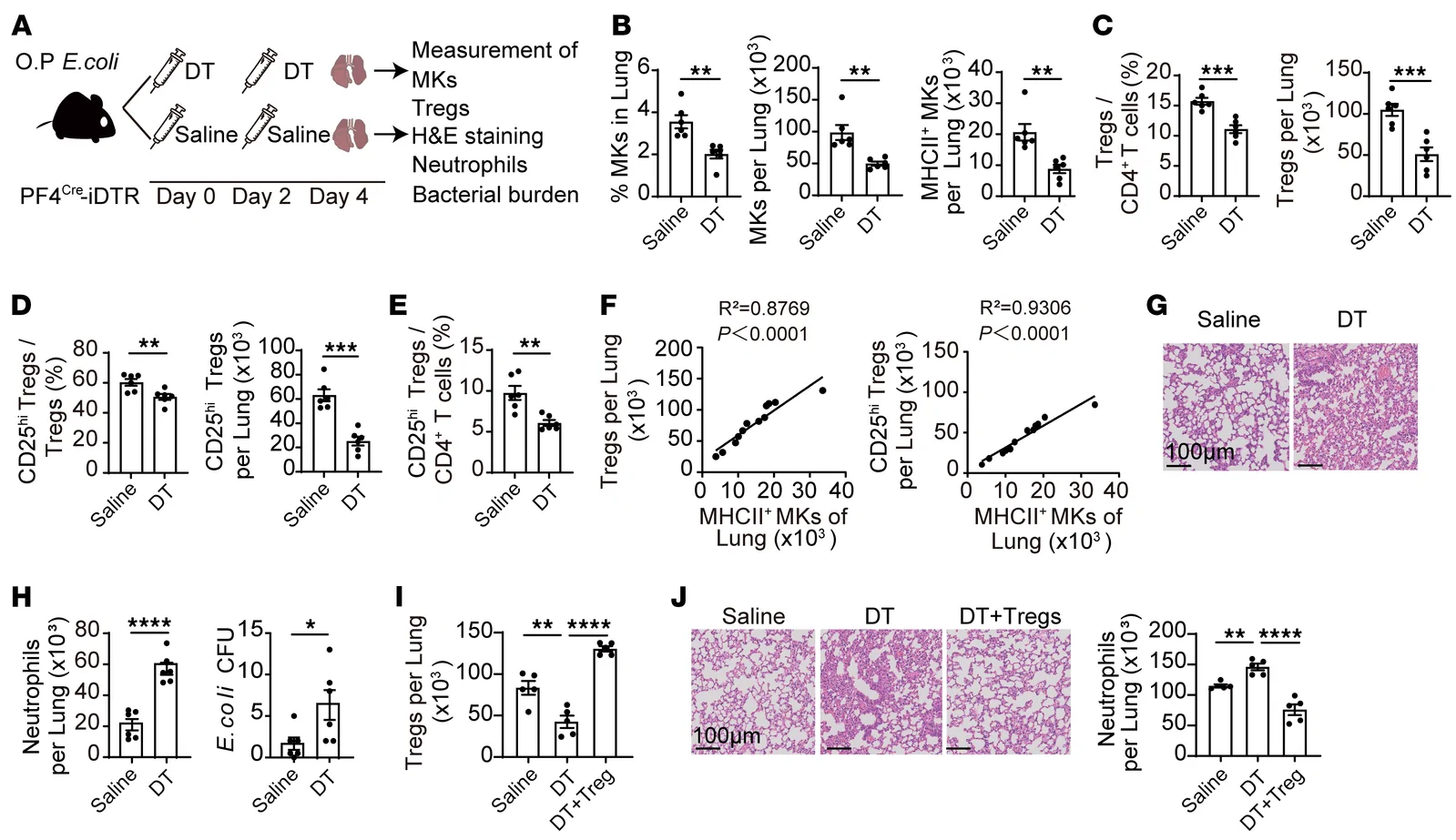
An APC-MK–Treg axis supports pulmonary inflammatory control during postpeak bacterial pneumonia. (**A**) Schematic illustration of the lung MK depletion strategy during the late stage of pneumonia. (**B**) Flow cytometry analysis of MK ratios (left), MK numbers (middle), and MHCII^+^ MK numbers (right) of control versus DT treatment mice. (**C**) Flow cytometry analysis of ratios (left) and numbers (right) of Tregs in control versus DT treatment mice. (**D**) Flow cytometry analysis of ratios (left) and numbers (right) of CD25^hi^ Treg in control versus DT treatment mice. (**E**) Flow cytometry analysis of CD25^hi^ Treg ratios in CD4^+^ T cells in control versus DT treatment mice. (**F**) Correlation analysis of MHCII^+^ MK numbers with Treg numbers (left) and CD25^hi^ Treg numbers (right) in the lungs of DT-treated mice (R^2^ = 0.8769 and 0.9306, respectively; *P* < 0.0001 for both). (**G**) Representative H&E-stained lung sections from control and DT-treated mice. Scale bar: 100 μm. (**H**) Flow cytometric quantification of pulmonary neutrophils in control and DT-treated mice (left); analysis of lung bacterial burden in control and DT-treated mice (right). (**I**) Flow cytometric quantification of pulmonary Tregs in control mice, DT-treated mice, and DT-treated mice receiving adoptive Treg transfer. (**J**) Representative H&E-stained lung sections (left) and flow cytometric quantification of pulmonary neutrophils (right) in control, DT-treated, and DT-treated mice receiving adoptive Treg transfer. Scale bar: 100 μm. Data are presented as mean ± SEM from 4 independent biological experiments for **B**–**E**, 3 independent experiments for **H**, and 2 independent experiments for **I** and **J**. Statistical analyses were performed using 2-tailed unpaired Student’s *t* test for **B**–**E** and **H**, linear regression analysis for **F**, and 1-way ANOVA followed by Tukey’s multiple-comparison test for **I** and **J**. *P < 0.05, **P < 0. 01, ***P < 0.001, ****P < 0.0001. Representative H&E-stained lung sections from at least 2 independent experiments, with similar results.

## Version 1 09/29/2026

In-Press Preview

## Version 2 11/16/2026

Electronic publication

## References

[1] Mazharian A, Senis YA (2025). Defining and harnessing the megakaryocyte/platelet checkpoint. Mol Cell Biol.

[2] Machlus KR, Italiano JE (2013). The incredible journey: From megakaryocyte development to platelet formation. J Cell Biol.

[3] Roberts HR (2006). A cell-based model of thrombin generation. Semin Thromb Hemost.

[4] Xu XR (2016). Platelets are versatile cells: New discoveries in hemostasis, thrombosis, immune responses, tumor metastasis and beyond. Crit Rev Clin Lab Sci.

[5] Tober J (2007). The megakaryocyte lineage originates from hemangioblast precursors and is an integral component both of primitive and of definitive hematopoiesis. Blood.

[6] Potts KS (2014). A lineage of diploid platelet-forming cells precedes polyploid megakaryocyte formation in the mouse embryo. Blood.

[7] Iturri L (2021). Megakaryocyte production is sustained by direct differentiation from erythromyeloid progenitors in the yolk sac until midgestation. Immunity.

[8] Xu MJ (2001). Evidence for the presence of murine primitive megakaryocytopoiesis in the early yolk sac. Blood.

[9] Palis J (2016). Hematopoietic stem cell-independent hematopoiesis: emergence of erythroid, megakaryocyte, and myeloid potential in the mammalian embryo. FEBS Lett.

[10] Davenport P (2020). Changes in megakaryopoiesis over ontogeny and their implications in health and disease. Platelets.

[11] Asquith NL (2024). The bone marrow is the primary site of thrombopoiesis. Blood.

[12] Malara A, Balduini A (2024). Megakaryocytes in the lung: guests or ghosts?. Blood.

[13] Lefrançais E (2017). The lung is a site of platelet biogenesis and a reservoir for haematopoietic progenitors. Nature.

[14] Livada AC (2024). Long-lived lung megakaryocytes contribute to platelet recovery in thrombocytopenia models. J Clin Invest.

[15] Boilard E, Machlus KR (2021). Location is everything when it comes to megakaryocyte function. J Clin Invest.

[16] Gelon L (2022). Occurrence and role of lung megakaryocytes in infection and inflammation. Front Immunol.

[17] Slayton WB (2002). The spleen is a major site of megakaryopoiesis following transplantation of murine hematopoietic stem cells. Blood.

[18] Valet C (2022). Sepsis promotes splenic production of a protective platelet pool with high CD40 ligand expression. J Clin Invest.

[19] Bruns I (2014). Megakaryocytes regulate hematopoietic stem cell quiescence through CXCL4 secretion. Nat Med.

[20] Zhao M (2014). Megakaryocytes maintain homeostatic quiescence and promote post-injury regeneration of hematopoietic stem cells. Nat Med.

[21] Heazlewood SY (2013). Megakaryocytes co-localise with hemopoietic stem cells and release cytokines that up-regulate stem cell proliferation. Stem Cell Res.

[22] Lan W (2024). A subset of megakaryocytes regulates development of hematopoietic stem cell precursors. EMBO J.

[23] Migliaccio AR (2021). A novel megakaryocyte subpopulation poised to exert the function of HSC niche as possible driver of myelofibrosis. Cells.

[24] Finney BA (2012). CLEC-2 and Syk in the megakaryocytic/platelet lineage are essential for development. Blood.

[25] Serrano N (2012). Megakaryocytes promote hepatoepithelial liver cell development in E11.5 mouse embryos by cell-to-cell contact and by vascular endothelial growth factor A signaling. Hepatology.

[26] Cunin P, Nigrovic PA (2019). Megakaryocytes as immune cells. J Leukoc Biol.

[27] Wang H (2021). Decoding human megakaryocyte development. Cell Stem Cell.

[28] Liu C (2021). Characterization of cellular heterogeneity and an immune subpopulation of human megakaryocytes. Adv Sci (Weinh).

[29] Wang J (2022). CXCR4^high^ megakaryocytes regulate host-defense immunity against bacterial pathogens. Elife.

[30] Tilburg J (2023). Spatial transcriptomics of murine bone marrow megakaryocytes at single-cell resolution. Res Pract Thromb Haemost.

[31] Li JJ (2024). Differentiation route determines the functional outputs of adult megakaryopoiesis. Immunity.

[32] Sun S (2021). Single-cell analysis of ploidy and the transcriptome reveals functional and spatial divergency in murine megakaryopoiesis. Blood.

[33] Xia M (2026). A machine learning-based megakaryocyte identification system uncovers resident organs, markers, and functional diversity. Dev Cell.

[34] Migliaccio AR, Hoffman R (2021). An outline of the outset of thrombopoiesis in human embryos at last. Cell Stem Cell.

[35] Vainchenker W, Raslova H (2021). The megakaryocyte: a cell with 3 faces as a mythic god?. Blood.

[36] Guo K (2024). The many faces of the megakaryocytes and their biological implications. Curr Opin Hematol.

[37] Tilburg J (2022). Don’t you forget about me(gakaryocytes). Blood.

[38] Chen SJ (2023). Ex vivo manufacturing of platelets: beyond the first-in-human clinical trial using autologous iPSC-platelets. Int J Hematol.

[39] Puhm F (2023). Diversity of megakaryocytes. Arterioscler Thromb Vasc Biol.

[40] Liu C (2021). The heterogeneity of megakaryocytes and platelets and implications for ex vivo platelet generation. Stem Cells Transl Med.

[41] Koupenova M (2022). Platelet and megakaryocyte roles in innate and adaptive immunity. Circ Res.

[42] Singh SV (2025). Immune and inflammatory properties of megakaryocytes. Cells.

[43] Li Y (2024). Immunological face of megakaryocytes. Front Med.

[44] Davenport P (2022). Fetal vs adult megakaryopoiesis. Blood.

[45] Li J (2023). Desialylated platelet clearance in the liver is a novel mechanism of systemic immunosuppression. Research (Wash D C).

[46] Li C (2024). Thrombocytopenia independently leads to changes in monocyte immune function. Circ Res.

[47] Karakas D, Ni H (2024). Unveiling platelets as immune regulatory cells. Circ Res.

[48] Qiu J (2024). Lung megakaryocytes engulf inhaled airborne particles to promote intrapulmonary inflammation and extrapulmonary distribution. Nat Commun.

[49] Pariser DN (2021). Lung megakaryocytes are immune modulatory cells. J Clin Invest.

[50] Köhler A (2011). G-CSF-mediated thrombopoietin release triggers neutrophil motility and mobilization from bone marrow via induction of Cxcr2 ligands. Blood.

[51] Zufferey A (2017). Mature murine megakaryocytes present antigen-MHC class I molecules to T cells and transfer them to platelets. Blood Adv.

[52] Guo L (2021). Platelet MHC class I mediates CD8^+^ T-cell suppression during sepsis. Blood.

[53] Chapman LM (2012). Platelets present antigen in the context of MHC class I. J Immunol.

[54] Kang HK (2012). Megakaryocyte progenitors are the main APCs inducing Th17 response to lupus autoantigens and foreign antigens. J Immunol.

[55] Finkielsztein A (2015). Human megakaryocyte progenitors derived from hematopoietic stem cells of normal individuals are MHC class II-expressing professional APC that enhance Th17 and Th1/Th17 responses. Immunol Lett.

[56] Fontana MF (2015). JUNB is a key transcriptional modulator of macrophage activation. J Immunol.

[57] McCowan J (2021). The transcription factor EGR2 is indispensable for tissue-specific imprinting of alveolar macrophages in health and tissue repair. Sci Immunol.

[58] Gombart AF (2003). Regulation of neutrophil and eosinophil secondary granule gene expression by transcription factors C/EBP epsilon and PU.1. Blood.

[59] Shahrin NH (2016). Conditional knockout mice demonstrate function of Klf5 as a myeloid transcription factor. Blood.

[60] Schebesta A (2007). Transcription factor Pax5 activates the chromatin of key genes involved in B cell signaling, adhesion, migration, and immune function. Immunity.

[61] Affandi AJ (2019). Low RUNX3 expression alters dendritic cell function in patients with systemic sclerosis and contributes to enhanced fibrosis. Ann Rheum Dis.

[62] Hamlett I (2008). Characterization of megakaryocyte GATA1-interacting proteins: the corepressor ETO2 and GATA1 interact to regulate terminal megakaryocyte maturation. Blood.

[63] Wang H (2018). MEIS1 Regulates hemogenic endothelial generation, megakaryopoiesis, and thrombopoiesis in human pluripotent stem cells by targeting TAL1 and FLI1. Stem Cell Reports.

[64] Vo KK (2017). FLI1 level during megakaryopoiesis affects thrombopoiesis and platelet biology. Blood.

[65] Poscablo DM (2024). An age-progressive platelet differentiation path from hematopoietic stem cells causes exacerbated thrombosis. Cell.

[66] Stolla MC (2019). regulates age-dependent differences in murine platelet function. Blood Adv.

[67] Maurya P (2023). Transfusion of adult, but not neonatal, platelets promotes monocyte trafficking in neonatal mice. Arterioscler Thromb Vasc Biol.

[68] Rossaint J (2021). Platelets orchestrate the resolution of pulmonary inflammation in mice by T reg cell repositioning and macrophage education. J Exp Med.

[69] Ma L (2015). CD8^+^ T cells are predominantly protective and required for effective steroid therapy in murine models of immune thrombocytopenia. Blood.

[70] Li S (2014). A naturally occurring CD8(+)CD122(+) T-cell subset as a memory-like Treg family. Cell Mol Immunol.

[71] Yeung AK (2020). Lung megakaryocytes display distinct transcriptional and phenotypic properties. Blood Adv.

[72] Elahi S (2013). Immunosuppressive CD71^+^ erythroid cells compromise neonatal host defence against infection. Nature.

[73] Xu C (2022). Single-cell transcriptomic analysis identifies an immune-prone population in erythroid precursors during human ontogenesis. Nat Immunol.

[74] He H (2020). Single-cell transcriptome analysis of human skin identifies novel fibroblast subpopulation and enrichment of immune subsets in atopic dermatitis. J Allergy Clin Immunol.

[75] Elyada E (2019). Cross-species single-cell analysis of pancreatic ductal adenocarcinoma reveals antigen-presenting cancer-associated fibroblasts. Cancer Discov.

[76] Geldhof V (2022). Single cell atlas identifies lipid-processing and immunomodulatory endothelial cells in healthy and malignant breast. Nat Commun.

[77] Yeung AK, Murphy GJ (2025). Breathing life into the hematopoietic potential of the lung. Blood.

[78] Conrad C (2025). Decoding functional hematopoietic progenitor cells in the adult human lung. Blood.

[79] Dobin A (2013). STAR: ultrafast universal RNA-seq aligner. Bioinformatics.

[80] Satija R (2015). Spatial reconstruction of single-cell gene expression data. Nat Biotechnol.

[81] Aran D (2019). Reference-based analysis of lung single-cell sequencing reveals a transitional profibrotic macrophage. Nat Immunol.

