# Supplementary material for: Immune subtypes of megakaryocytes orchestrate inflammatory and regulatory responses to pulmonary infection via Treg crosstalk
Source: J Clin Invest. 2026 Sep 29;136(22):e201621. doi: 10.1172/JCI201621 (PMC13767634; doi:10.1172/JCI201621)
Supplement: Supplementary file 1 [file jci-136-201621-s059.pdf]

Supplemental Figure 1

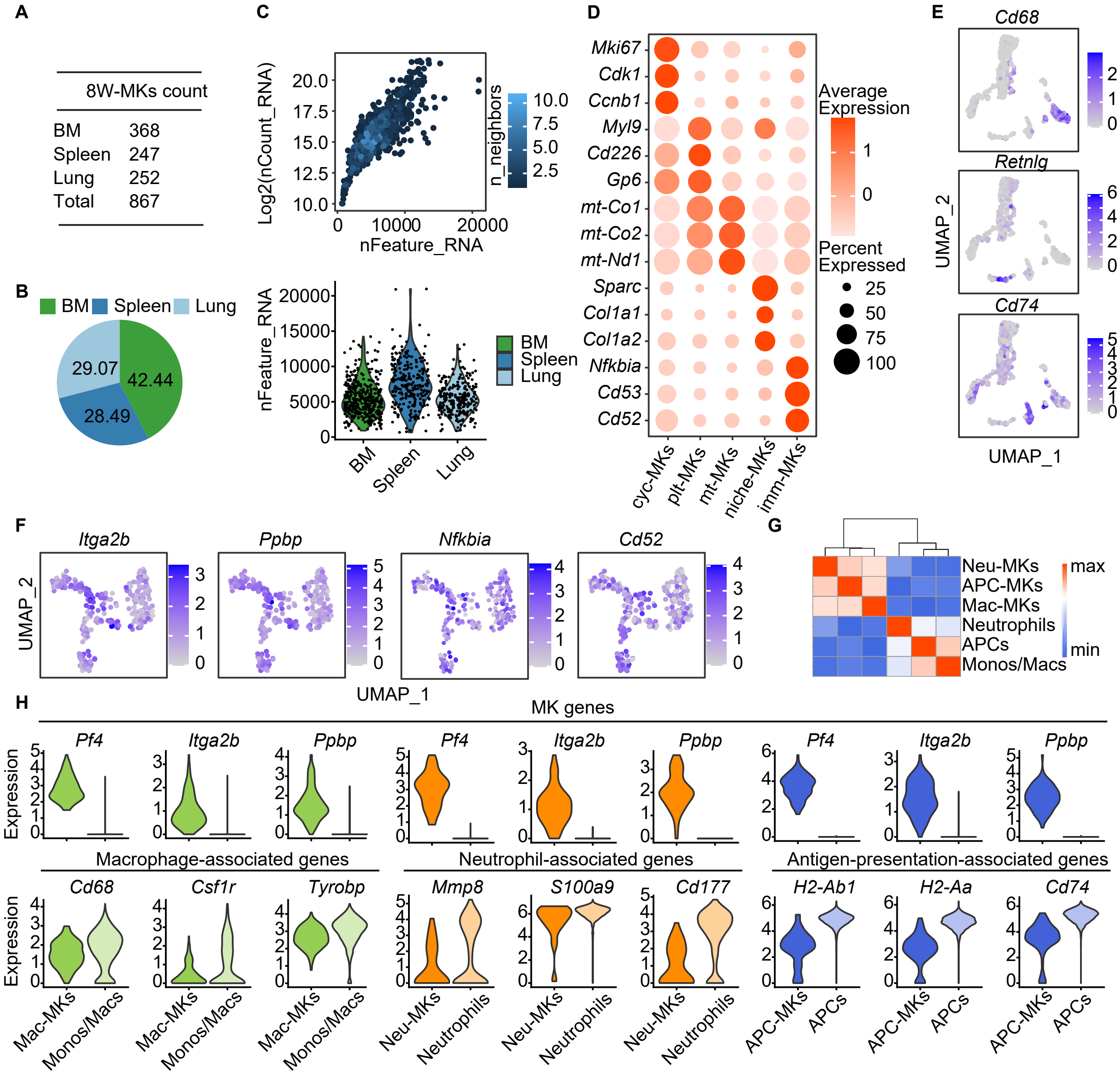

Supplemental Figure 1. Integrated transcriptomic analysis of mouse adult megakaryocytes across multiple organs, related to Figure 1.

A. Table summarizing the numbers of MKs from the bone marrow, spleen, and lung of adult mice included in the single-cell RNA-sequencing analysis.

B. Pie chart showing the proportions of MKs from the bone marrow, spleen, and lung of adult mice included in the single-cell RNA-sequencing analysis.

C. Scatter plot showing the relationship between nFeature and log-transformed nCount values (top) and violin plots showing the numbers of detected genes in MKs from each organ (bottom).

D. Dot plot showing representative marker genes for the five MK subpopulations.

E. Feature plots showing the expression of *Cd68*, *Retnlg*, and *Cd74* in adult mouse MKs.

F. Feature plots showing the expression of MK-lineage and general immune-cell markers in adult mouse immune MKs.

G. Heatmap showing transcriptomic correlations between canonical immune-cell populations—monocytes/macrophages, neutrophils, and antigen-presenting cells—and their corresponding immune MK subtypes—Mac-MKs, Neu-MKs, and APC-MKs.

H. Violin plots showing the expression levels of MK-lineage and immune-lineage signature genes in macrophages, neutrophils, antigen-presenting cells, Mac-MKs, Neu-MKs, and APC-MKs.

Supplemental Figure 2

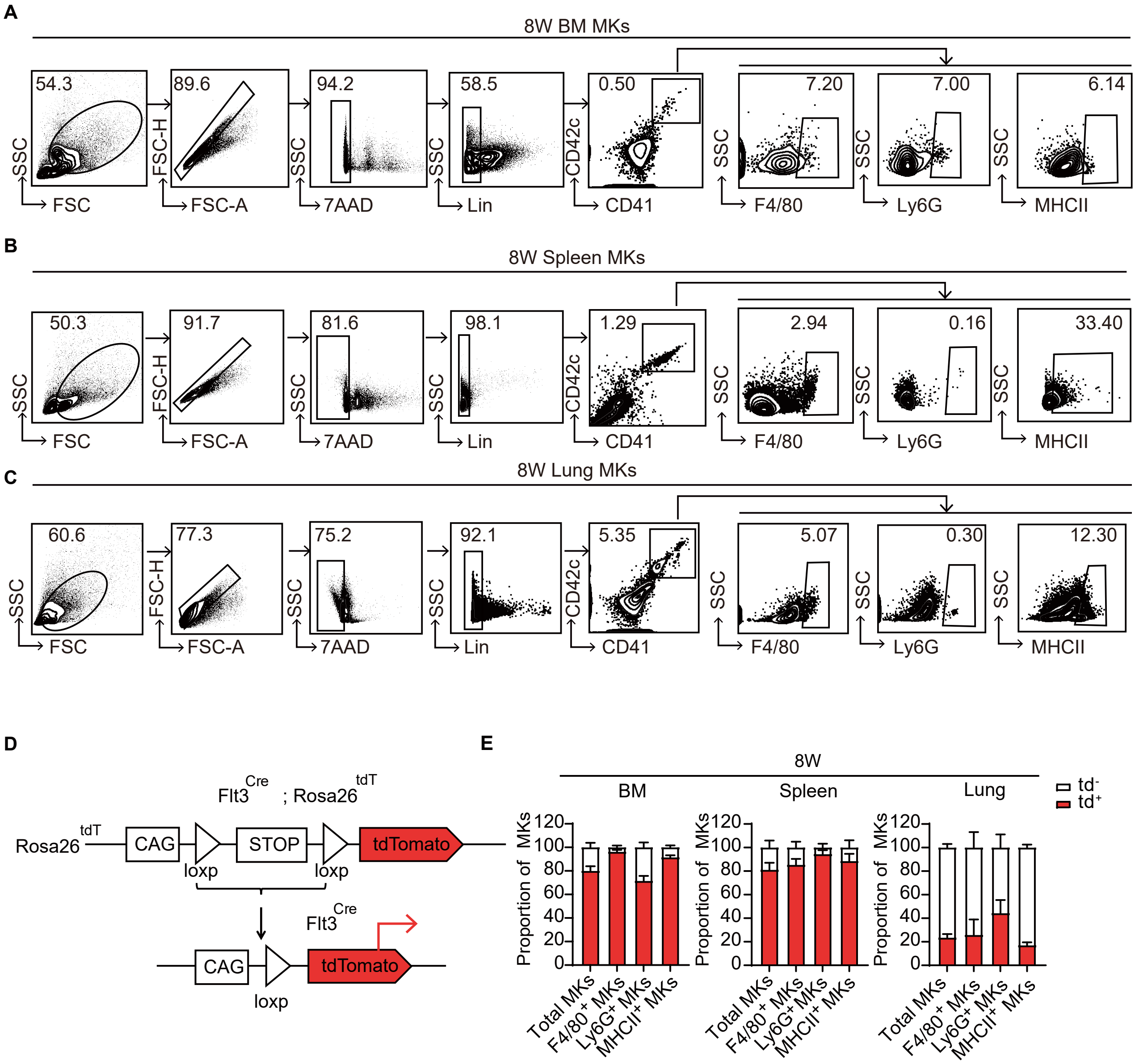

Supplemental Figure 2. Flow cytometry gating strategy and Flt3-lineage tracing of immune-associated MK phenotypes, related to Figure 2.

- **A.** Representative flow cytometry plots illustrating the gating strategy used to identify immune MK subtypes in the bone marrow.
- **B.** Representative flow cytometry plots illustrating the gating strategy used to identify immune MK subtypes in the spleen.
- **C.** Representative flow cytometry plots illustrating the gating strategy used to identify immune MK subtypes in the lung.
- **D.** Schematic of the $Flt3^{Cre}$; $Rosa26^{tdTomato}$ lineage-tracing model used to distinguish Flt3-lineage-traced and Flt3-lineage-untraced MKs.
- **E.** Flow cytometric analysis of $tdTomato^{+}$ and $tdTomato^{-}$ fractions among total MKs, $F4/80^{+}$ MKs, $Ly6G^{+}$ MKs, and $MHCII^{+}$ MKs in adult bone marrow, spleen, and lung.

Data are presented as mean ± SEM from 2 independent biological experiments for E.

Supplemental Figure 3

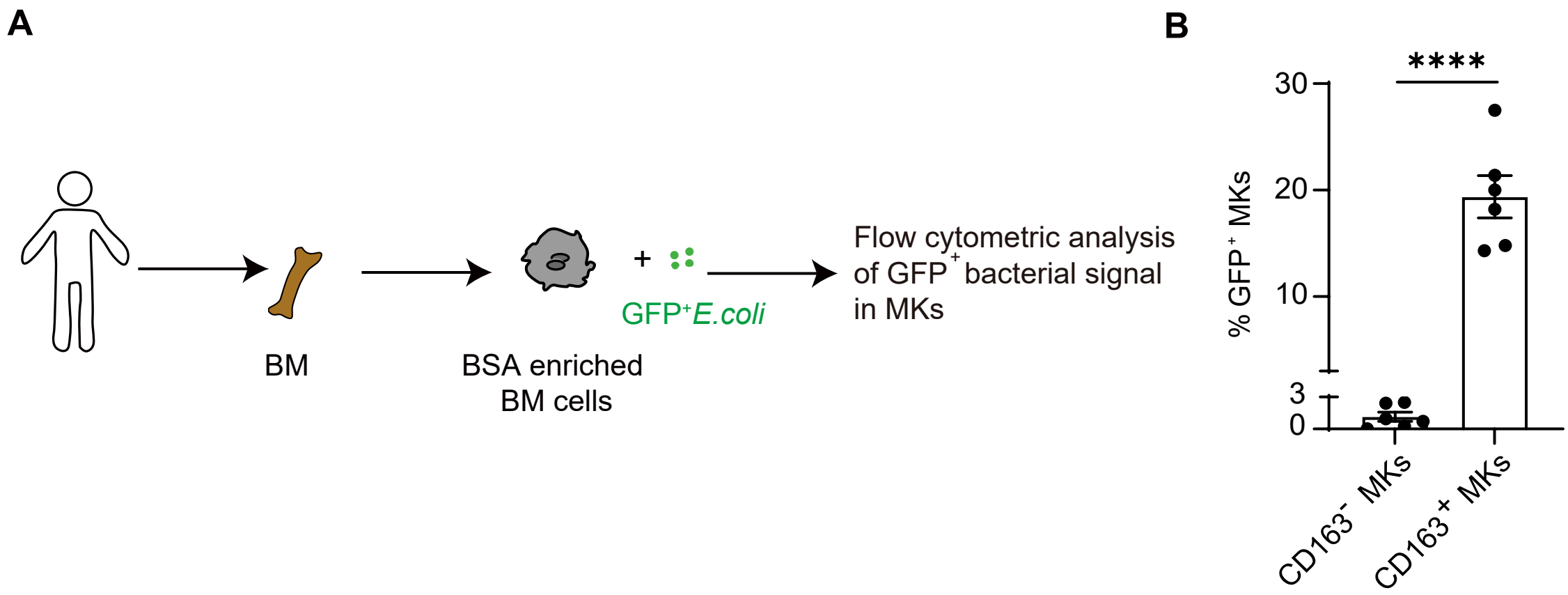

Supplemental Figure 3. Human bone marrow CD163$^+$ Mac-MKs exhibit enriched GFP$^+$ *E. coli*-associated signals *in vitro*, related to Figure 3.

A. Schematic of the *in vitro* co-incubation assay using human bone marrow samples and GFP$^+$ *E. coli*, followed by flow-cytometric analysis of GFP$^+$ signals in human MKs.

B. Flow cytometric analysis of GFP-positive bacterial signals in CD163$^-$ and CD163$^+$ MKs. n = 6 per group.

Data are presented as mean ± SEM from 5 independent biological experiments for B. Statistical analyses were performed using 2-tailed unpaired Student's *t* test for B. ****$P < 0.0001$.

Supplemental Figure 4

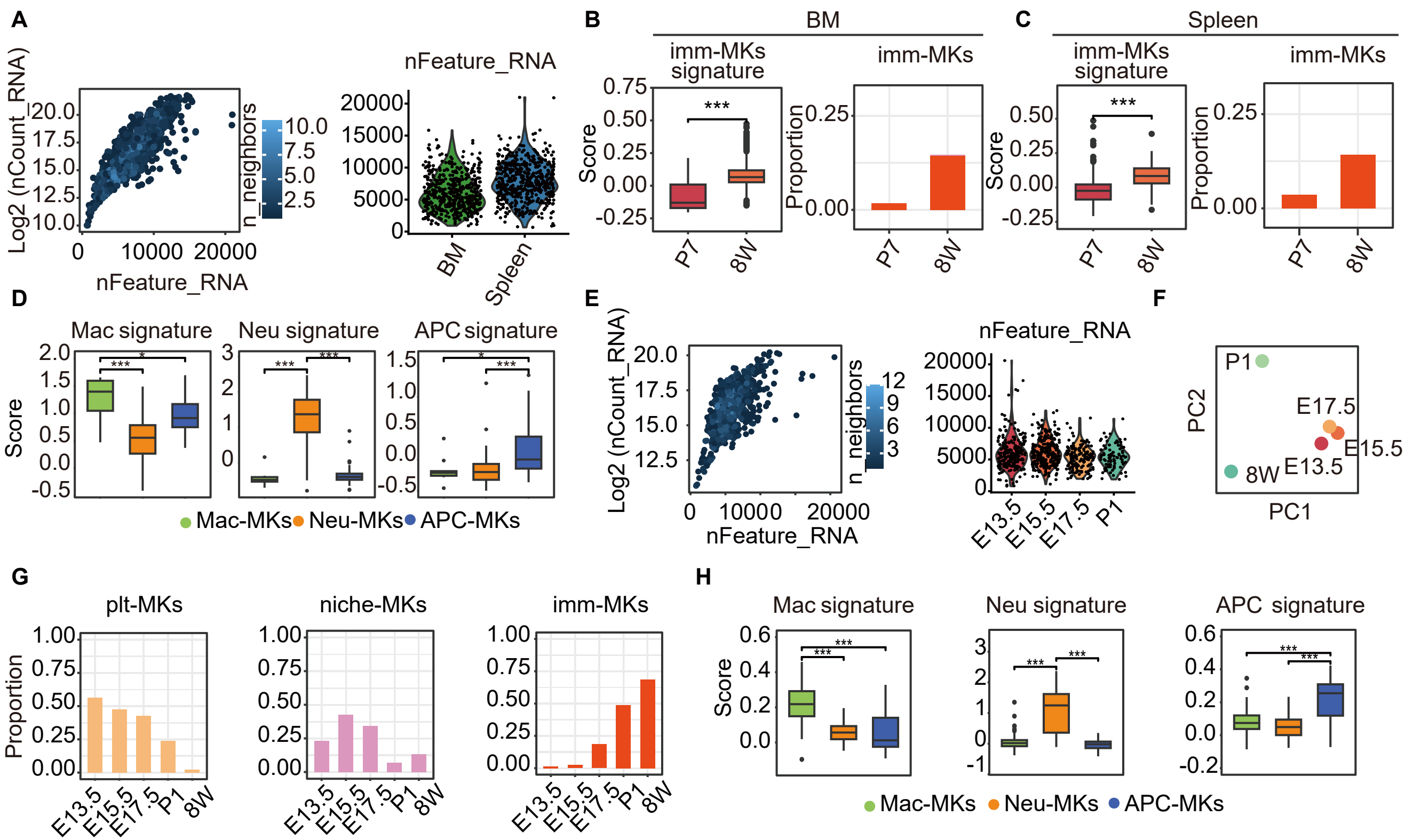

Supplemental Figure 4. Developmental remodeling of immune MK subpopulations in bone marrow, spleen, and lung, related to Figure 4.

A. Scatter plot showing nFeature vs. Log (nCount) in P7 bone marrow and spleen MKs (left), and violin plot showing gene counts in P7 bone marrow and spleen MKs (right).

B. Boxplot showing changes in immune signatures of bone marrow MKs across development (Supplemental Table 2A) (left). The median value is indicated by a horizontal line, and the box represents the first and third quartiles. Bar chart showing developmental changes in immune subpopulation proportions of MKs in bone marrow (right).

C. Boxplot showing changes in immune signatures of splenic MKs across development (Supplemental Table 2A) (left). The median value is indicated by a horizontal line, and th box represents the first and third quartiles. Bar chart showing developmental changes in immune subpopulation proportions of MKs in spleen (right).

D. Boxplots showing immune signature scores of different immune MK subtypes (Supplemental Table 3A). The median is indicated by a horizontal line, and boxes represent the first and third quartiles.

E. Scatter plot showing nFeature vs. Log (nCount) in lung MKs across timepoints (left), and violin plot showing gene counts in lung MKs across timepoints (right).

F. PCA plot and clustering results for lung MKs at different timepoints.

G. Bar plots showing temporal proportions of platelet-related (plt), niche-related MK and imm-MK subtypes.

H. Boxplots showing immune signature scores of different immune MK subtypes (Supplemental Table 3A). The median is indicated by a horizontal line, and boxes represent the first and third quartiles.

Statistical analyses were performed using using two-sided Wilcoxon rank-sum tests and adjusted for multiple comparisons using the Benjamini-Hochberg method for D and H; *$P < 0.05$, ***$P < 0.001$.

Supplemental Figure 5

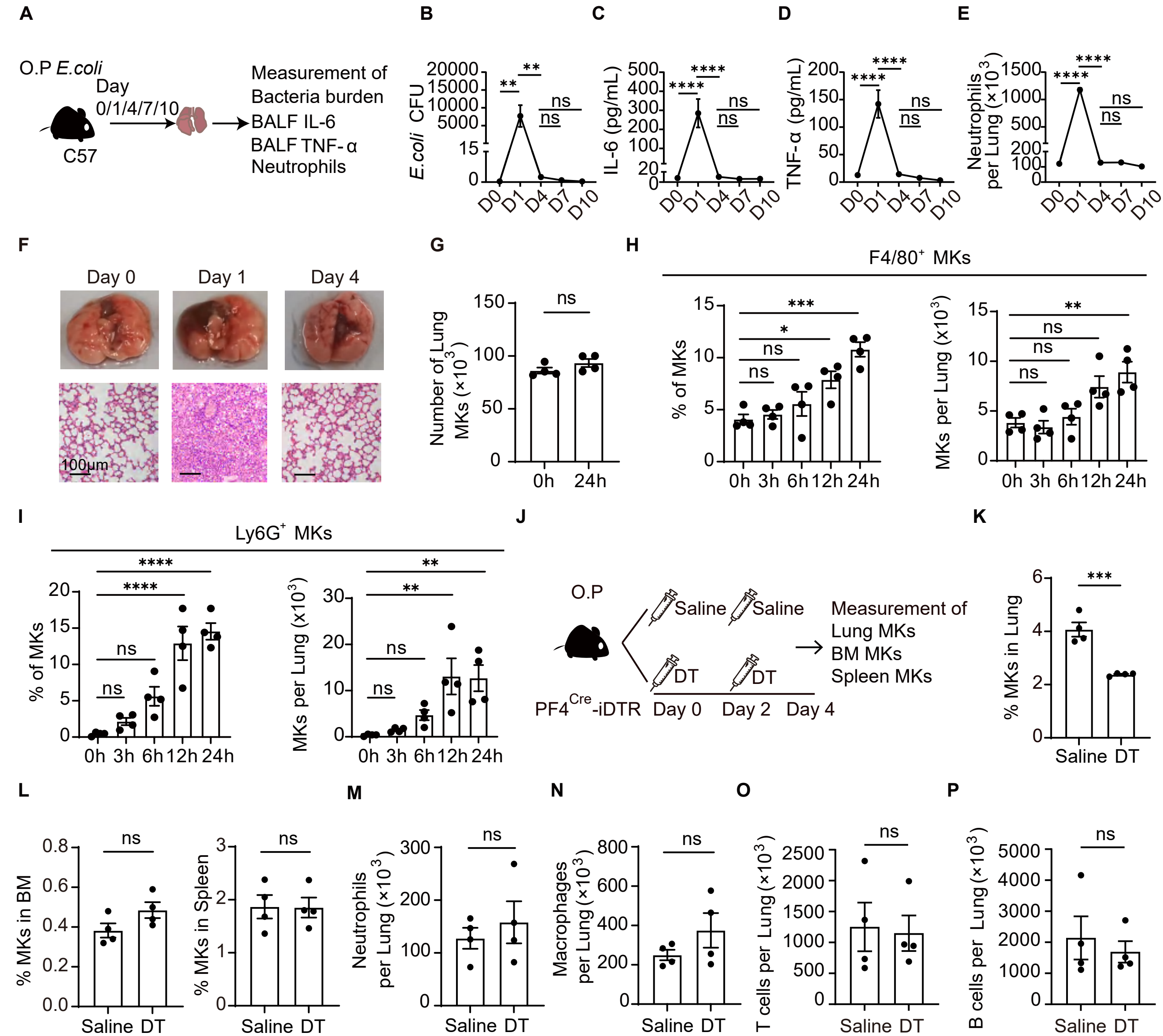

Supplemental Figure 5. Time-course characterization of the *E. coli* pneumonia model and control analyses for early lung MK depletion, related to Figure 5.

A. Schematic illustration of the experimental timeline for *E. coli*-induced pneumonia in mice.
B. Quantification of residual bacteria in the lungs at the indicated time points after infection.
C. Changes in IL-6 concentrations in bronchoalveolar lavage fluid (BALF) at the indicated time points after infection.
D. Changes in TNF-α concentrations in BALF at the indicated time points after infection.
E. Changes in pulmonary neutrophil numbers at the indicated time points after infection.
F. Representative gross images (top) and H&E-stained sections (bottom) of lungs collected at the indicated time points after infection. Scale bar, 100 μm.
G. Flow cytometric quantification of lung MK number during the early stage of pneumonia.
H. Flow cytometric analysis of changes in the proportions and number of pulmonary Mac-MKs at the indicated early time points after infection.
I. Flow cytometric analysis of changes in the proportions and number of pulmonary Neu-MKs at the indicated early time points after infection.
J. Schematic illustration of oropharyngeal DT administration for specific depletion of lung MKs in $PF4^{Cre}$-iDTR mice.
K. Proportions of lung MKs in control and DT-treated mice.
L. Proportions of bone marrow and splenic MKs in control and DT-treated mice.
M. Flow cytometric quantification of pulmonary neutrophils in control and DT-treated mice.
N. Flow cytometric quantification of pulmonary macrophages in control and DT-treated mice.
O. Flow cytometric quantification of pulmonary T cells in control and DT-treated mice.
P. Flow cytometric quantification of pulmonary B cells in control and DT-treated mice.

Data are presented as mean ± SEM. For B, C, and E, n = 6 biological replicates at D0, D1, D4, D7, and D10. For D, n = 6 biological replicates at D0, D1, D4, and D7, and n = 3 biological replicates at D10. G represents 3 independent biological experiments, H and I represent 4 independent biological experiments, and K–P represent 2 independent biological experiments. Statistical analyses were performed using 2-tailed unpaired Student's *t* test for G and K-P and 1-way ANOVA followed by Tukey's multiple comparison test for B-E, H, and I. ns, not significant, $^{*}P < 0.05$, $^{**}P < 0.01$, $^{***}P < 0$.

001, ****$P < 0.0001$. Representative gross images and H&E-stained lung sections from at least 2 independent experiments, with similar results.

Supplemental Figure 6

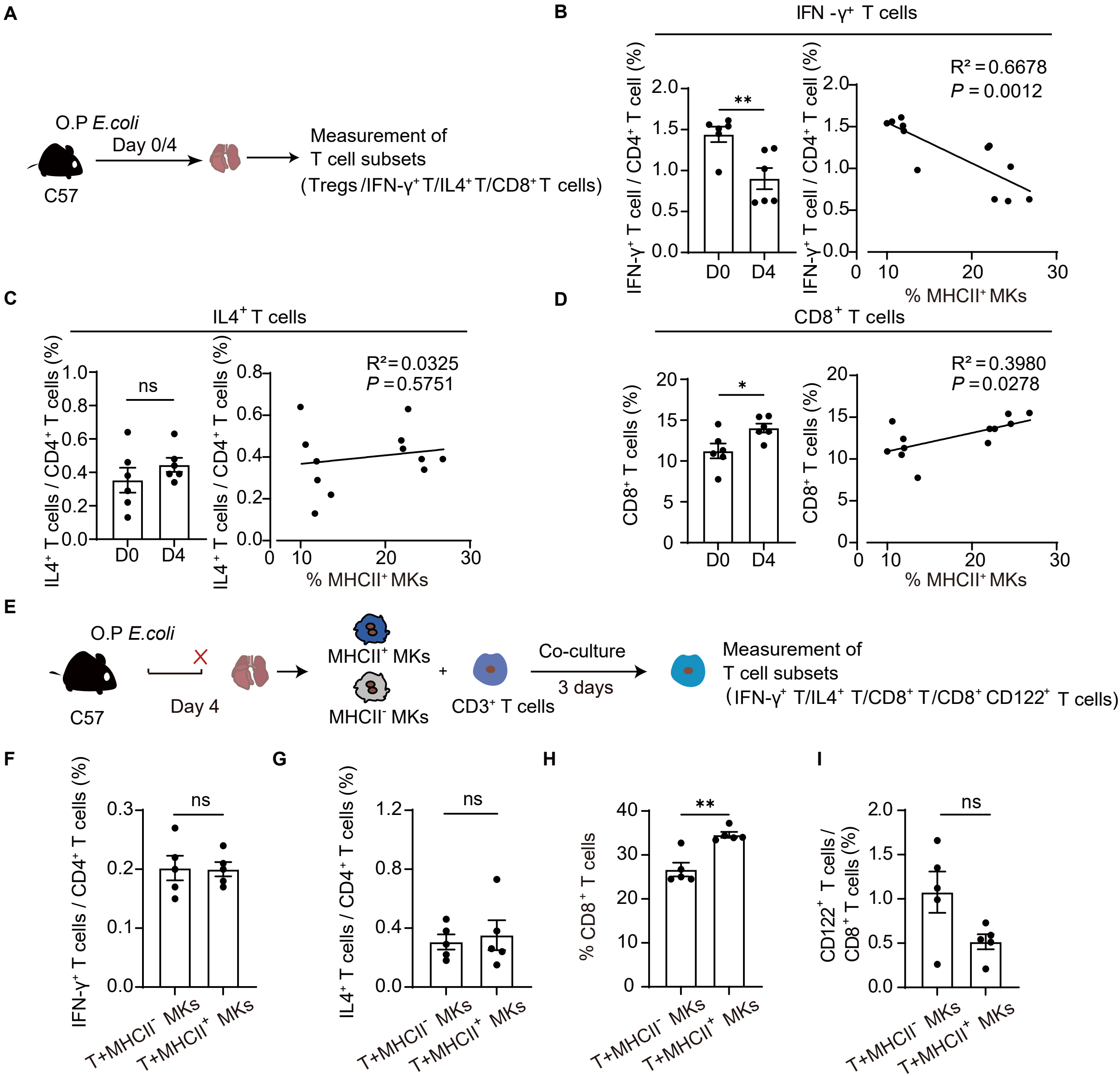

Supplemental Figure 6. Additional T-cell readouts show non-uniform changes during pneumonia as well as following co-culture with MHCII$^+$ MKs, related to Figure 6.

A. Schematic illustration of the experimental strategy used to assess T-cell subsets during the course of pneumonia.

B. Changes in the proportion of IFN-γ^+ T cells at the indicated time points after infection (left) and correlation between the proportions of pulmonary MHCII$^+$ MKs and IFN-γ^+ T cells in mice with pneumonia (right; $R^2 = 0.6678$, $P = 0.0012$).

C. Changes in the proportion of IL-4$^+$ T cells at the indicated time points after infection (left) and correlation between the proportions of pulmonary MHCII$^+$ MKs and IL-4$^+$ T cells in mice with pneumonia (right; $R^2 = 0.0325$, $P = 0.5751$).

D. Changes in the proportion of CD8$^+$ T cells at the indicated time points after infection (left) and correlation between the proportions of pulmonary MHC II$^+$ MKs and CD8$^+$ T cells in mice with pneumonia (right; $R^2 = 0.3980$, $P = 0.0278$).

E. Schematic illustration of the *in vitro* co-culture of T cells with APC-MKs.

F. Flow cytometric analysis of the proportions of IFN-γ^+ T cells following co-culture with MHCII$^-$ or MHCII$^+$ MKs.

G. Flow cytometric analysis of the proportions of IL-4$^+$ T cells following co-culture with MHCII$^-$ or MHCII$^+$ MKs.

H. Flow cytometric analysis of the proportions of CD8$^+$ T cells following co-culture with MHCII$^-$ or MHCII$^+$ MKs.

I. Flow cytometric analysis of the proportions of CD8$^+$ CD122$^+$ T cells following co-culture with MHCII$^-$ or MHCII$^+$ MKs.

Data are presented as mean ± SEM from 4 independent biological experiments for B-D, and 3 independent biological experiments for F-I. Statistical analyses were performed using 2-tailed unpaired Student's *t* test for B-D and F-I, and linear regression analysis for B-D. ns, not significant, $^*P < 0.05$, $^{**}P < 0.\ 01$.

Supplemental Figure 7

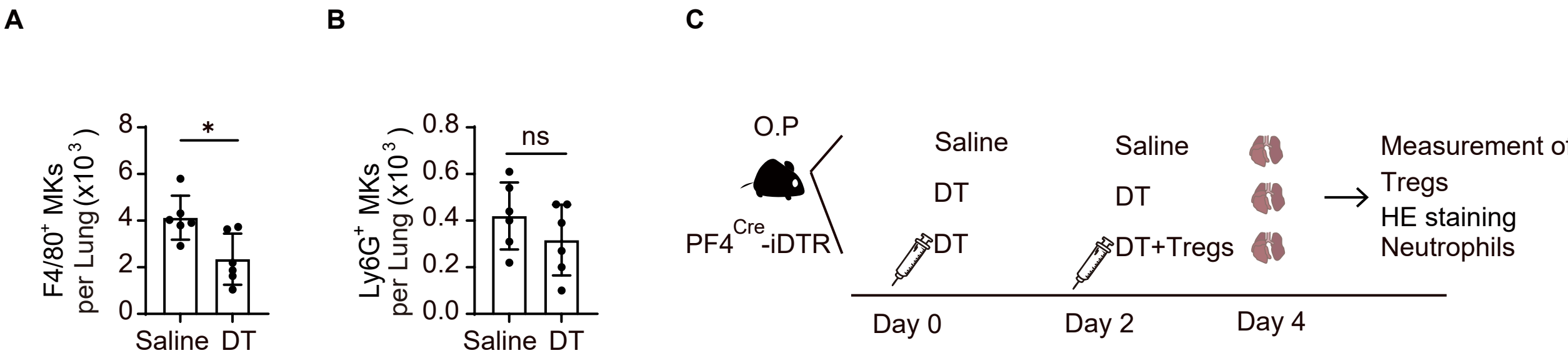

Supplemental Figure 7. Changes in F4/80$^+$ and Ly6G$^+$ MKs after lung MK depletion and Treg supplementation strategy, related to Figure 7.

A. Flow cytometric quantification of Mac-MKs in control and DT-treated mice.
B. Flow cytometric quantification of Neu-MKs in control and DT-treated mice.
C. Schematic illustration of the adoptive Treg transfer strategy in the pneumonia model.
Data are presented as mean ± SEM from 4 independent biological experiments for A and B. Statistical analyses were performed using 2-tailed unpaired Student's *t* test for A and B. ns, not significant, *$P < 0.05$.

Supplemental Table 4. KEY RESOURCES

REAGENT or RESOURCE	*SOURCE*	*IDENTIFIER*
Antibodies		
FITC anti-mouse CD41 (Clone MWReg30)	*BD Bioscience*	*Cat# 553848;*<br>*RRID: AB_395085*
FITC anti-mouse CD41 Antibody	*Biolegend*	*Cat# 133904;*<br>*RRID: AB_2129746*
APC anti-mouse CD41 (Clone MWReg30)	*Biolegend*	*Cat# 133914;*<br>*RRID: AB_11125581*
PE anti-mouse CD41 Antibody	*Biolegend*	*Cat# 133906;*<br>*RRID: AB_2129745*
Alexa Fluor® 700 anti-mouse CD41 Antibody	*Biolegend*	*Cat# 133926;*<br>*RRID: AB_2572130*
FITC Mouse Anti-Human CD41a	*BD Bioscience*	*Cat# 555466;*<br>*RRID: AB_395858*
Antibody for In Vivo Mouse Platelet Labeling （CD42C）	*Emfret*	*Cat# X488*<br>*RRID: AB_2890921*
Antibody for In Vivo Mouse Platelet Labeling （CD42C）	*Emfret*	*Cat# X649*<br>*RRID: AB_2861336*
APC Mouse Anti-Human CD42b	*BD Bioscience*	*Cat# 551061;*<br>*RRID: AB_398486*
PE/Cyanine7 anti-mouse CD3 Antibody	*Biolegend*	*Cat# 100220;*<br>*RRID: AB_1732057*
Alexa Fluor® 647 anti-mouse CD3 Antibody	*Biolegend*	*Cat# 100209;*<br>*RRID: AB_389323*
APC/Cyanine7 anti-mouse CD3 Antibody	*Biolegend*	*Cat# 100222;*<br>*RRID: AB_2242784*
FITC anti-mouse CD3 Antibody	*Biolegend*	*Cat# 100204;*<br>*RRID: AB_312661*
PE/Cyanine7 anti-mouse/human CD45R/B220 Antibody	*Biolegend*	*Cat# 103222;*<br>*RRID: AB_313005*
PE/Cyanine7 anti-mouse/human CD11b Antibody	*Biolegend*	*Cat# 101216;*<br>*RRID: AB_312799*
PE/Cyanine7 anti-mouse TER-119/Erythroid Cells Antibody	*Biolegend*	*Cat# 116222;*<br>*RRID: AB_2281408*
APC/Cyanine7 anti-mouse I-A/I-E Antibody	*Biolegend*	*Cat# 107628;*<br>*RRID: AB_2069377*
APC anti-mouse I-A/I-E Antibody	*Biolegend*	*Cat# 107614;*<br>*RRID: AB_313329*
In Vivo MAb anti-mouse MHC Class II (I-A/I-E)	*BioXCell*	*Cat# M5/114;*<br>*RRID: AB_3265910*

BD Pharmingen™ PE Rat Anti-Mouse Ly-6G	*BD bioscience*	*Cat# 551461;*<br>*RRID: AB_394208*
APC anti-mouse Ly-6G Antibody	*Biolegend*	*Cat# 127614;*<br>*RRID: AB_2227348*
Brilliant Violet 510™ anti-mouse Ly-6G Antibody	*Biolegend*	*Cat# 127633;*<br>*RRID: AB_2562937*
PE/Cyanine5 anti-mouse F4/80 Antibody	*Biolegend*	*Cat# 123112;*<br>*RRID: AB_893482*
PE anti-mouse F4/80 Antibody	*Biolegend*	*Cat# 123110;*<br>*RRID: AB_893486*
APC anti-mouse F4/80 Antibody	*Biolegend*	*Cat# 123116;*<br>*RRID: AB_893481*
PE/Cyanine7 anti-human CD163 Antibody	*Biolegend*	*Cat# 326514;*<br>*RRID: AB_2565245*
APC/Cyanine7 anti-human HLA-DR Antibody	*Biolegend*	*Cat# 307618;*<br>*RRID: AB_493586*
PE anti-human CD177 Antibody	*Biolegend*	*Cat# 315806;*<br>*RRID: AB_2564281*
FITC anti-mouse CD4 Antibody	*Biolegend*	*Cat# 100406;*<br>*RRID: AB_312691*
APC anti-mouse CD4 Antibody	*Biolegend*	*Cat# 100412;*<br>*RRID: AB_312697*
PE/Cyanine7 anti-mouse CD19 Antibody	*Biolegend*	*Cat# 152418;*<br>*RRID: AB_2927870*
APC/Cyanine7 anti-mouse CD25 Antibody	*Biolegend*	*Cat# 101918;*<br>*RRID: AB_2650982*
PE anti-mouse CD25 Antibody	*Biolegend*	*Cat# 102008;*<br>*RRID: AB_312857*
PE/Cyanine7 anti-mouse CD8a Antibody	*Biolegend*	*Cat# 100722;*<br>*RRID: AB_312761*
PE/Cyanine5 anti-mouse CD8a Antibody	*Biolegend*	*Cat# 100710;*<br>*RRID: AB_312749*
PE anti-mouse CD122 (IL-2Rb) Antibody	*Biolegend*	*Cat# 105906;*<br>*RRID: AB_2125736*
PE/Cyanine7 anti-mouse IL-4 Antibody	*Biolegend*	*Cat# 504118;*<br>*RRID: AB_10898116*
FOXP3 Monoclonal Antibody (FJK-16s), PE	*eBioscience™*	*Cat# 12-5773-82;*<br>*RRID: AB_465936*
Alexa Fluor 647 anti-mouse Foxp3 Antibody	*Biolegend*	*Cat# 126408;*<br>*RRID: AB_1089115*
TGF-beta 1, 2, 3 Antibody	*R&D Systems*	*Cat# MAB1835-100;*<br>*RRID: AB_357931*

PE anti-mouse IFN-r Antibody	*Biolegend*	*Cat# 163504;*<br>*RRID: AB_2890730*
Goat anti-Rabbit IgG (H+L) Cross-Adsorbed Secondary Antibody, Alexa Fluor™ 594	*Invitrogen*	*Cat# A-11012;*<br>*RRID: AB_141359*
Rat IgG2a kappa Isotype Control	*Invitrogen*	*Cat# 12-4321-83;*<br>*RRID: AB_470053*
Rat IgG2b kappa Isotype Control	*eBioscience*	*Cat# 14-4031-85;*<br>*RRID: AB_470100*
APC Rat IgG2a, κ Isotype Ctrl Antibody	*Biolegend*	*Cat# 400512;*<br>*RRID: AB_2814702*
APC/Cyanine7 Rat IgG1, κ Isotype Ctrl Antibody	*Biolegend*	*Cat# 400422;*<br>*RRID: AB_830905*
APC/Cyanine7 Rat IgG2b, κ Isotype Ctrl Antibody	*Biolegend*	*Cat# 400624;*<br>*RRID: AB_326566*
PE Rat IgG2a, κ Isotype Ctrl Antibody	*Biolegend*	*Cat# 400508;*<br>*RRID: AB_326530*
PE Rat IgG2b, κ Isotype Ctrl Antibody	*Biolegend*	*Cat# 400636;*<br>*RRID: AB_893669*
APC/Cyanine7 Mouse IgG2a, κ Isotype Ctrl Antibody	*Biolegend*	*Cat# 400230;*<br>*RRID: AB_326477*
PE/Cyanine7 Mouse IgG1, κ Isotype Ctrl Antibody	*Biolegend*	*Cat# 400126;*<br>*RRID: AB_326448*
PE/Cyanine7 Mouse IgG2b, κIsotype Ctrl Antibody	*Biolegend*	*Cat# 653820;*<br>*RRID: AB_3083421*
PE/Cyanine7 Rat IgG1, κ Isotype Ctrl Antibody	*Biolegend*	*Cat# 400416;*<br>*RRID: AB_326522*
PE Mouse IgG1, κ Isotype Ctrl (FC) Antibody	*Biolegend*	*Cat# 400114;*<br>*RRID: AB_326435*
Brilliant Violet 510™ Rat IgG2a, κ Isotype Ctrl Antibody	*Biolegend*	*Cat# 400547;*<br>*RRID: AB_3097655*
PE/Cyanine5 Rat IgG2a, κ Isotype Ctrl Antibody	*Biolegend*	*Cat# 400510;*<br>*RRID: AB_326531*
PE anti-mouse/rat CD62P (P-selectin) Antibody	*Biolegend*	*Cat# 148306;*<br>*RRID: AB_2565275*
Anti-mouse CD16/32 Antibody	*Biolegend*	*Cat# 156604;*<br>*RRID: AB_2783138*
TruStain FcX™ (anti-mouse CD16/32) Antibody	*Biolegend*	*Cat# 101320;*<br>*RRID: AB_2783138*
CD28 Monoclonal Antibody (37.51), Functional Grade, eBioscience™	*Invitrogen*	*Cat# 16-0281-86;*<br>*RRID: AB_468923*
CD3 Monoclonal Antibody (OKT3), Functional Grade, eBioscience™	*Invitrogen*	*Cat# 16-0031-86;*<br>*RRID: AB_468849*
Biological samples		

Bone Marrow, Spleen, Lung samples from mouse embryos and adult mice	This paper	This paper
Bone Marrow samples from Human	This paper	This paper
Chemicals, peptides, and recombinant proteins		
Fetal Bovine Serum	Gibco	Cat# 16000044
Bovine Serum Albumin (BSA)	Solarbio	Cat# A8020
Normal saline	Solarbio	Cat# 7647-14-5
Hoechst 33342	Solarbio	Cat# C0031
Zombie Violet Fixable Viability Kit	Biolegend	Cat# 42311
7AAD	BD bioscience	Cat# 559925
7AAD	Biolegend	Cat# 420403
DAPI	Solarbio	Cat# C0060
Trucount™ Trucount Absolute Counting Tubes	BD	Cat# 340334
Sodium citrate	Vocas	Cat# 68-04-2
Ethylene Diamine Tetraacetic Acid (EDTA, 0.5M)	Solarbio	Cat# E1170
NaCl	Kerme	Cat# 7647-14-5
Triton-X-100	Biotech	Cat# P0096
Sucrose	Solarbio	Cat# S8271
4% Paraformaldehyde (PFA) tissue fixation solution	Solarbio	Cat# P1110
Phosphate Buffer Saline (PBS)	Beyotime	Cat# C0221A
Penicillin/Streptomycin/Amphotericin B, sterile solution (100×)	Solarbio	Cat# P7630
Gelatin	Solarbio	Cat# G8061
Tryptone	Oxoid	Cat# LP0042B
Yeast Extract	Oxoid	Cat# LP0021B
Red Blood Cell Lysis Buffer	Solarbio	Cat# R1010
Collagenase □	Sigma-Aldrich	Cat# C2674
DN25	Sigma-Aldrich	Cat# 9003-98-9
RNase Inhibitor (40U/μl)	Invitrogen	Cat# AM2684
Nuclease-free water	Peprotech	Cat# 315-14
dNTP (10 mM)	Fermentas	Cat# R0193
10% Triton X-100	Sigma-Aldrich	Cat# T8787
10% Neutral Buffered Formalin	Sigma-Aldrich	Cat# HT501128
0.1M DTT	Invitrogen	Cat# 18064-071
Betaine (5M)	Sigma-Aldrich	Cat# 61962
MgCl2 (1M)	Sigma-Aldrich	Cat# M8266
KAPA HiFi HotStart ReadyMix (2x)	KAPA	Cat# Kk2602
1M Tris-HCL（pH7.5）	Sigma-Aldrich	Ca # T2944
Dynabeads® MyOne™ Streptavidin C1	Invitrogen	Cat# 65001
AMPure XP beads	Beckman	Cat# A63882

SuperScript II reverse transcriptase(200 U/μl)	*Invitrogen*	*Cat# 18064-071*
Superscript II first-strand buffer(5x)	*Invitrogen*	*Cat# 18064-071*
Mouse IL-2 Recombinant Protein	*PeproTech*	*Cat# 212-12-20UG*
Thrombin	*Sigma-Aldrich*	*Cat# T9326-150UN*
Critical commercial assays		
KAPA Hyper Prep Kits with PCR Library Amplification/Illumina series	*KAPA*	*Cat# KK8504*
Qubit dsDNA HS Assay Kit	*Invitrogen*	*Cat# Q32854*
DNA clean&Collection Kit	*ZYMO*	*Cat# D4014*
Agilent High Sensitivity DNA Kit	*Agilent*	*Cat# 5067-4626*
NEB Next Multiplex Oligos for Illumina (Index Primers Set 1)	*NEB*	*Cat# E7335L*
EasySep™mouse T cell Isolation Kit	*Stemcell*	*Cat# 19851*
Mouse $CD4^+$ $CD25^+$ Regulatory T Cell Isolation Kit II	*Stemcell*	*Cat# 18783*
eBioscience™ Foxp3 / Transcription Factor Staining Buffer Set	*eBioscience*	*Cat# 00-5523-00*
Deposited data		
Single-cell sequencing data of mouse megakaryocytes	*This study*	*National Omics Data Encyclopedia: OED01031223, OED01031224, and OED01042029*
Single-cell sequencing data of human megakaryocytes	*Wang et al[1]; Liu et al[2]*	*National Omics Data Encyclopedia: GSE144024 and OEP001150*
Experimental models: Organisms/strains		
Mouse: C57BL/6J	*Beijing HFK Bioscience Co., Ltd*	*Cat# 11001A*
Software and algorithms		
R (version: 4.1.0)	*The R Foundation*	*http://www.r-project.org/*
STAR (version 2.7.10a)	*Dobin, et al[3]*	*http://code.google.com/p/rna-star/.*
ggpubr (version 0.6.0)	*R package*	*https://github.com/kassambara/ggpubr*
ggplot2 (version 3.4.1)	*R package*	*https://ggplot2.tidyverse.org/*
pheatmap (version 1.0.12)	*R package*	*https://cran.r project.org/web/packages/pheatmap/index.html*

Seurat (version 4.1.1)	*Satija et al*[4]	*https://satijalab.org/seurat/index.html*
SingleR(version v1.0.1)	*R package*[5]	*https://github.com/dviraran/SingleR*
FlowJo(version 10)	*TreeStar*	*http://www.flowjo.com*
GraphPad Prism(version 8.0.1)	*GraphPad Software*	*http://www.graphpad.com*
Imaris(version 9.0.1)	*Bitplane, Oxford Instrument Company*	*http://imaris.oxinst.com*
NDP.view2	*Hamamatsu Photonics K.K. (EU, Japan, UK, USA)*	*http://www.hamamatsu..com*
IDEAS software(Version 6.2)	*Amnis, Luminex Corporation*	*http://www.amnis.com*
